# 3D Bioprinting of Prevascularized Full-Thickness Gelatin-Alginate Structures with Embedded Co-Cultures

**DOI:** 10.3390/bioengineering9060242

**Published:** 2022-05-31

**Authors:** Bastian Böttcher, Astrid Pflieger, Jan Schumacher, Berit Jungnickel, Karl-Heinz Feller

**Affiliations:** 1 Institute for Microsystem and Precision Engineering, Ernst-Abbe University of Applied Science Jena, 07745 Jena, Germany; bastian.boettcher@eah-jena.de (B.B.); pflieger.astrid@gmail.com (A.P.); jan_schumacher_2208@t-online.de (J.S.); 2 Department of Cell Biology, Institute of Biochemistry and Biophysics, Faculty of Biological Sciences, Friedrich Schiller University Jena, 07745 Jena, Germany; berit.jungnickel@uni-jena.de

**Keywords:** bioprinting, gelatin-alginate hydrogel, prevascularization, co-culture, rheological characterization, coaxial extrusion

## Abstract

The use of bioprinting allows the creation of complex three-dimensional cell laden grafts with spatial placements of different cell lines. However, a major challenge is insufficient nutrient transfer, especially with the increased size of the graft causing necrosis and reduced proliferation. A possibility to improve nutrient support is the integration of tubular structures for reducing diffusion paths. In this study the influence of prevascularization in full-thickness grafts on cell growth with a variation of cultivation style and cellular composition was investigated. To perform this, the rheological properties of the used gelatin-alginate hydrogel as well as possibilities to improve growth conditions in the hydrogel were assessed. Prevascularized grafts were manufactured using a pneumatic extrusion-based bioprinter with a coaxial extrusion tool. The prevascularized grafts were statically and dynamically cultured with a monoculture of HepG2 cells. Additionally, a co-culture of HepG2 cells, fibroblasts and HUVEC-TERT2 was created while HUVEC-TERT2s were concentrically placed around the hollow channels. A static culture of prevascularized grafts showed short-term improvements in cell proliferation compared to avascular grafts, while a perfusion-based culture showed improvements in mid-term cultivation times. The cultivation of the co-culture indicated the formation of vascular structures from the hollow channels toward avascular areas. According to these results, the integration of prevascular structures show beneficial effects for the in vitro cultivation of bioprinted grafts for which its impact can be increased in larger grafts.

## 1. Introduction

For medical, pharmaceutical and cosmetical purposes, it is necessary to develop organ-near cell constructs of complex natures. For several years, this was performed by means of two-dimensional structures, providing the possibility to substitute animal tests [1,2,3]. However, it was shown in the last years that two-dimensional (2D) structures are in most cases too far from in vivo conditions of the human body/organs. It is well known that cells, which grow in traditional two-dimensional (2D) cell culture monolayers, lack physiologically relevant environmental conditions. They mostly exhibit drastic differences of their physical and biochemical properties in comparison to intact biological systems. Therefore, much effort was placed into the development of three-dimensional structures [4] in order to image complex behavior in reality. It was unfortunately shown that the usability of such constructs is limited also in this case mainly due to structural limitations abd also due to the limited lifetime of such constructs [5,6]. A major cause is the decline in diffusion with increasing distance to the surrounding nutrient medium in 3D-constructs, leading to a shortage of oxygen and acidification [7]. Bioprinting is a powerful tool to create layer-by-layer generated three-dimensional cell-laden constructs from a computer-generated file. This allows the creation of distinct 3D structures with a spatial arrangement of different cell lines. These three-dimensional cell constructs made of cell-laden hydrogels manufactured via bioprinting offer a promising tool to investigate cellular activity due to its improved resemblance of in vivo conditions compared to traditional 2D cell culture [8,9,10]. The most widespread form of bioprinting is extrusion-based bioprinting that uses pneumatic driven forces or a stamp to promote material flow [11,12]. Due to the modular design of many extrusion-based bioprinters, customization according to the desired application is possible. A versatile bioprinting tool is a coaxial extruder that allows printing two materials simultaneously in a core/shell manner. Coaxial extrusion has been used for a coaxial placement of two different cell lines. Taymour et al. created a co-culture with core-located fibroblasts and shell-located HepG2 cells encapsulated in a alginate-methylcellulose-based hydrogel [13]. Wang et al. mimicked the glioma microenvironment using coaxial extrusion. Thereby, they encapsulated GSC23 cells in alginate and located them in the shell compartment while U118 cells were extruded through the core-nozzle [14]. Another example of coaxial printing to create spatially defined co-cultures is provided by Li et al. RSC96 cells were encapsulated in an alginate gel and extruded as the shell while a cell suspension of NE4C-cells was located at the core [15]. These examples show the possibility for spatially orientating different cell lines in proximity while enabling the generation of cell specific environments by extruding different cell-laden materials through each nozzle. However, another promising application of coaxial extrusion is the formation of hollow channels. Mostly, hollow channels were created without coaxial extrusion by printing sacrificial materials that were removed in the post-printing procedure such as Pluronic F127 [16], gelatin [17] or carbohydrate glasses [18]. Using coaxial extrusion, hollow channels can be generated with and without sacrificial materials using a crosslinker. Hollow channel generation without sacrificial material can be achieved using a crosslinker for the shell-located hydrogel as a core material. A common combination is the usage of alginate as shell-material and calcium chloride as the crosslinker in the core-section [19,20]. Kim and Nam generated hollow channels using Pluronic F127 loaded with CaCl_2_ as sacrificial core-material while pre-crosslinked alginate was used as the shell. CaCl_2_-laden Pluronic F127 caused the post-crosslinking of alginate and was removed later [21]. The insertion of endothelial cells in the shell-compartment is a promising method for the fast generation of simplified vessels with an endothelium. Without coaxial extrusion, endothelial cells were delivered by injecting a cell suspension into the prevascularized network [16,17]. However, a major drawback of this method is the necessity to make a physical connection to the hollow channel for cell delivery. While it is appropriate when one complex prevascular network has to be seeded, it becomes more difficult when several independent channels are printed, especially when the channel’s diameter is small. This problematic procedure can be avoided if cell delivery is performed using coaxial extrusion. Gao et al. [22] used a triple coaxial extruder to produce cell-laden hollow fibers with endothelial cells and smooth muscle cells that were later implanted in mice. However, only single fibers were produced without further integration into an in vitro-based surrounding bioprinted graft that might show the formation of vascular structures. Additionally, the implementation of these channels into a full-thickness, pore-free graft could provide reduced diffusion paths, leading to improved nutrient delivery. Traditionally, bioprinted constructs are printed with artificial pores to reduce diffusion paths and improve cell viability. However, the gaps cannot be used for any cellular functions and remain unfunctional. Instead, using embedded hollow strands, the diffusion paths could be reduced in full-thickness constructs, and different cell lines with unique functionalities can be inserted. To evaluate the versatile possibilities of coaxially printed hollow strands in prevascularized constructs, mono- and co-cultures were bioprinted into prevascularized gelatin-alginate based constructs. To conduct this, a material characterization was performed with subsequent optimization in crosslinking and post-crosslinking for improving the cellular environment of three cell lines. After establishing the bioprinting process for prevascularized grafts, HepG2 cells were used in a monoculture to evaluate the potential for the improvement of the nutrient supply with coaxially printed hollow channels due to their high metabolic activity and the consequently increased need for nutrients. Furthermore, these grafts were additionally cultured in a 3D-printed reactor for assessing the positive effects of continuous cultivation. Finally, HUVEC and fibroblasts were added into the prevascularized grafts to evaluate the possibility for angiogenesis and the endothelialization of hollow tubes at static cultivation conditions.

## 2. Materials and Methods

### 2.1. Materials

Cell culture medium DMEM Low Glucose, DMEM F-12, Penicillin/Streptomycin, geneticin and FBS were obtained from Biowest (Nuaillé, France). Cell culture media MCDB 131 and rhEGF were purchased from PAN Biotech (Aidenbach, Germany). Hydrocortison, ascorbic acid, DAPI, DPBS, propidium iodide and gelatin type A (300 bloom) were obtained from Merck (Darmstadt, Germany). Heparin sodium salt was purchased from Serva (Heidelberg, Germany). Sodium alginate, calcium chloride, tri-sodium citrate-dihydrate, silicon oil, Roti-Immunoblock and L-glutamine were ordered from Carl Roth (Karlsruhe, Germany). Bovine pituitary extract was obtained from Thermo Fisher/Life Technologies (Darmstadt, Germany). Resazurin sodium salt was purchased at Alfa Aesar (War Hill, GA, USA). CD31 antibody was purchased from Becton Dickinson (Heidelberg, Germany). Calcein blue AM was purchased from AAT Bioquest (Sunnyvale, CA, USA). Cyclo-Olefin Copolymer COC 5013 granulat (TOPAS Advanced Polymers GmbH, Frankfurt am Main, Germany) was a friendly gift from 3D Schilling GmbH. COC slides were purchased from Microfluidic Chipshop (Jena, Germany). Microbial transglutaminase (mTGM) was obtained from Ajinomoto (Hamburg, Germany).

### 2.2. Preparation of Hydrogels

Gelatin and sodium alginate powders were weighted and transferred into a tube, homogenized with PBS and vortexed. Afterward, the gel was heated in a steam cooker for 60 min followed by a repeated homogenization step. The gelatin/alginate gel was cooled down and stored at room temperature. For bioprinting applications, combinations of 6% (*w*/*v*) gelatin and 2% (*w*/*v*) alginate and 6% (*w*/*v*) gelatin 4% alginate in PBS and 7% (*w*/*v*) gelatin solved in 100 mM CaCl_2_ were prepared.

### 2.3. Rheologic Investigations

For rheological investigations, a rheometer MCR 502 (Anton Paar, Graz, Austria) with a plate–plate measurement system was used. Before measurement, the temperature was set to 25 °C to achieve consistent conditions for all measurements. To prevent dehydration of samples during measurement, silicon oil was pipetted on the measurement system after equilibration of the measurement distance. To determine the influence of the hydrogel components, a DoE (Design of Experiment) was performed according to a Latin Hypercube Design with a concentration range of gelatin from 5% (*w*/*v*) to 10% (*w*/*v*) and alginate from 2% (*w*/*v*) to 10% (*w*/*v*). Within this DoE, the complex modulus at 25 °C with different deformations at ω = 10/s and the temperature behavior of the gels while heating from 25 to 37 °C with a deformation of 1% at ω = 10/s were investigated. In addition, the time to reach the sol/gel point after cooling down from 37 °C to 25 °C was determined with a deformation of 1% at ω = 10/s. To evaluate the complex modulus of the hydrogels used for bioprinting, a deformation-based measurement was performed at temperatures of 20 °C and 30 °C, which represented critical temperatures for the bioprinting process. The behavior of gels during the entire bioprinting process including preprocessing was investigated by rotational measurements. Thereby, a shear rate of 1/s was used to mark the relaxation behavior of the gel while a shear rate of 500/s was used to simulate the extrusion. However, the measurement times for each part were partially reduced compared to the actual bioprinting process due to the higher surface-volume-ratio in the measurement gap compared to bulk behavior.

### 2.4. Diffusion Behavior

To determine the diffusion behavior of molecules with a size similar to cytokines with the size of VEGF throughout the hydrogel, FITC-Dextran with a molecular weight of 20 kDa was used. A 6% (*w*/*v*) gelatin/2% (*w*/*v*) alginate hydrogel was heated and casted into a 3D-printed mold with an inserted needle. After cooling down, the needle was removed, creating a hollow channel. Afterward, the gel was transferred into a well plate for further crosslinking. Sodium citrate was added to samples, when the removal of alginate with respect to the diffusion capability was tested. After that, the FITC-dextran solution (100 µg/mL) was inserted into the channel, and image acquisition using an LSM 710 directly began. For the perfusion based experiment, the FITC-dextran solution was continuously added with a flow rate of 2.5 µL/min.

### 2.5. Cell Culture

HepG2 cells (CLS, Eppelheim, Germany) were cultivated using DMEM Low Glucose medium supplemented with 1% penicillin/streptomycin, 1% L-glutamine and 10% FBS in an incubator with 5% CO_2_ at 37 °C. Subcultivation was performed every 2–3 days by washing with PBS/EDTA followed by a five-minute incubation with trypsin/EDTA. HUVEC-TERT2 cells were obtained from Evercyte (Austria) and cultivated using MCDB 131 as basal medium supplemented with 10% FBS, 10 mM L-glutamine, 15 µg/mL bovine pituitary extract, 20 µg/mL genetincin, 50 µg/mL ascorbic acid, 0.75 IU/mL heparin sodium salt, 10 ng/mL rhEGF and 1 µg/mL hydrocortisone. Primary fibroblasts were isolated from human lung tumor tissue. Briefly, the tissue was mechanically dissected and agitated in a solution of 150 U/mL collagenase, 0.5 U/mL dispase and 7.5 U/mL Dnase in DMEM Low Glucose for 180 min at 37 °C. The won cell suspension was then filtered through a 100 µm cell strainer and incubated in an RBC lysis buffer for 5 min at room temperature prior to the washing and seeding of the cells in collagen-coated dishes. After several days of growth, the fibroblasts were separated from tumor cells by short incubation with accutase and subsequent seeded in a new culture dish. All primary fibroblasts were used with a passage number less than or equal to 10. Culture was performed using DMEM F-12 as a basal medium supplemented with 5% FBS, 2 mM l-glutamine and penicillin/streptomycin. Medium exchange for the printed samples was performed every 2–3 days. Co-Culture experiments were performed with the medium used for cultivation of HUVEC-TERT2 with a substitution of geneticin with penicillin/streptomycin.

### 2.6. Bioprinting Process

Bioprinting was performed using a pneumatic bioprinter BioScaffolder 3.2 (GeSiM, Radeberg, Germany). It was equipped with heatable cartridge holders for printing avascular structures. The used 10 cc cartridges (Vieweg, Kranzberg, Germany) were equipped with a 250 µm cylindrical nozzle (Vieweg, Kranzberg, Germany). In addition, a holder for the coaxial printing tool was equipped, facilitating the mountable coaxial printing tool that contains connection points for two cartridges. The coaxial tool was equipped with a 100 µm core nozzle and a 610 µm shell nozzle. Non-prevascularized 3D constructs were printed using a 6% gelatin 2% alginate hydrogel using a pressure of 60 kPa. Afterward, the samples were firstly crosslinked with a 20 mM CaCl_2_ solution for 10 min followed by an addition of 10 U/mL microbial transglutaminase (mTGM) for 60 min. After that, the constructs were rinsed with PBS and filled up with medium. The bioprinting of prevascularized constructs was performed in a three-step-process. Firstly, a base layer was printed with a cell-free 6% gelatin 2% alginate hydrogel. Afterward, the coaxial printing tool was used to print hollow strands. For this, a 6% gelatin 4% alginate hydrogel was used as shell material, while a CaCl_2_-laden 7% gelatin was used as the core material. The instant crosslinked strand was printed on all base layers in a meander-like pattern. In the last step, a cell-laden 6% gelatin 2% alginate hydrogel was printed between the coaxial printed strands. After printing, the samples were crosslinked with 100 mM CaCl_2_ for a short time and then mechanically separated by cutting the continuous printed coaxial strand at the border of the sample via a scalpel. After crosslinking with 20 mM CaCl_2_ for 10 min, the samples were stored for 30 min in cell culture medium in the incubator. This allowed the solubilization of gelatin and the possibility of its subsequent aspiration from the coaxial-printed channel. Then the samples were crosslinked with 10 U/mL transglutaminase for 60 min. After crosslinking, samples were rinsed with PBS and further cultivated in cell culture media. Depending on the experiment, different cell lines with specific cell concentrations were used. In general, the concentration of HepG2 was 1 × 10^7^ cells/mL hydrogel, HUVEC-TERT2 had a concentration of 5 × 10^6^ cells/mL hydrogel, and fibroblasts were used with a concentration of 2.5 × 10^6^ cells/mL. In prevascularized models, different constellations were prepared. In a HepG2 mono-culture, the shell compartment as well as the gel between the hollow strands were seeded with 1 × 10^7^ cells/mL each. In co-culture models, the shell compartment was always filled with HUVEC-TERT2, while the gel between the hollow strands was either laden with HepG2 or with HepG2 and fibroblasts. The treatment of the samples with sodium citrate was performed the next day after the bioprinting process. The parameters for experiments with prevascularized samples were a concentration of 100 mM sodium citrate and an incubation time of 5 min.

### 2.7. Manufacture and Set Up of the Bioreactor System

The manufacture of the bioreactor was performed using the high temperature extruder module of the BioScaffolder 3.2 that was loaded with COC 5013 granulate. Before printing, the granulate was molten at a cartridge temperature of 220 °C and a nozzle temperature of 240 °C for 45 min. Directly before printing, a COC slide was added as a printing substrate to form the base layer of the reactor. Directly before starting the printing and throughout the printing too, the substrate was heated. The generated G-Code of the CAD data was slightly modified to improve the printing quality of the chip. After printing, a silicon tube was added on each side of the connector and bonded with an adhesive to achieve a sealed connection. For later connections, Luer adapters were plugged on the silicon tubes. To seal the the reactor chamber, pressure-sensitive adhesive foils were used. For the geometric adaption of these foils, a laser structure was applied using Keyence ML-Z9520. When prevascularized constructs were cultured with the bioreactor, the constructs were first transferred into the chamber that was afterward sealed with pressure-sensitive adhesive foils followed by a manual filling of the reactor with medium via silicon tubes. The inlet side of the reactor was connected with a Safeflow-Adapter to the medium bottle. The outlet was connected with a sterile filter that was also connected with a Safeflow adapter, which had tubing to the waste bottle. Within this tubing path, a tube for pumping was integrated. Both reactor and medium bottle were placed into the incubator while the pump and waste bottle were placed outside. The continuous cultivation was performed at a flowrate of 25 µL/min.

### 2.8. Viability Analysis

To investigate the time-dependent viability of the cells, calcein-AM and propidium iodide staining for the visualization of live and dead cells was used. Briefly, cells were washed for 5 min in HBSS-buffer prior to the staining in calcein AM and PI (each 1 µM) for 15 min. Afterward, the constructs were washed three times for 5 min in HBSS again before microscopic imaging with a LSM 710 (Carl Zeiss Microscopy, Jena, Germany). A resazurin assay was used to quantify the viability over time. For this, a 50 µM solution of resazurin was added to the cells and incubated for 60 min in the incubator. Measurement was performed using a SPARK plate reader (TECAN, Männedorf, Switzerland) with a measurement wavelength of 555 nm and a reference wavelength of 605 nm.

### 2.9. Influence of the Crosslinker on Cell Viability

To evaluate the influence of used crosslinkers on cell viability, a DoE with a central composite design was performed. For this experiment, the hydrogel consisting of 6% gelatin and 2% alginate was used for all cell lines. In this setup, CaCl_2_ was added first to the bioprinted cell laden hydrogel followed by the second crosslinking step with transglutaminase. Thereby, the concentration and incubation time of CaCl_2_ and transglutaminase varied. The concentration of CaCl_2_ had a range from 20 mM to 100 mM, while transglumatinase was used from 1 U/mL to 10 U/mL. The incubation time of CaCl_2_ varied from 10 min to 60 min. For transglutaminase, incubation times from 1 h to 24 h were used. After crosslinking, sodium citrate was not added. All parameters concerning the bioprinting process and analysis were the same as before mentioned. The resazurin assay was performed one day and seven days after printing. The resulting signal difference of each sample was calculated and analyzed with Ansys OptiSlang (Weimar, Germany).

### 2.10. Immunofluorescence

Immunohistochemical staining was performed with an initial washing step with PBS followed by a treatment of Zamboni fixative for two hours. Afterward, three washing steps with each 20 min was performed. Subsequently, 0.1% Triton X100 was added for one hour for permeabilization. After repeated washing, the samples were blocked using a mixture of 2% BSA/10% Roti-Immunoblock. After three-hour incubation, the primary labeled antibodies were added and incubated overnight. To remove excess antibodies the next day, the samples were washed three times for one hour each. Afterward, DAPI was added for 15 min, and unbound DAPI was removed by washing the samples three times for 20 min each. Afterward, the samples were stored in a mounting medium.

### 2.11. Statistical Analysis

Statistical Analysis was performed using Origin 2015 (OriginLab, Northampton, MA, USA). Significance analysis was determined using two-sample *t*-test including a check of standard distribution. If the sample showed no standard distribution, the Mann–Whitney U test was used.

## 3. Results

### 3.1. Investigation of Rheological Properties

**Figure 1:**
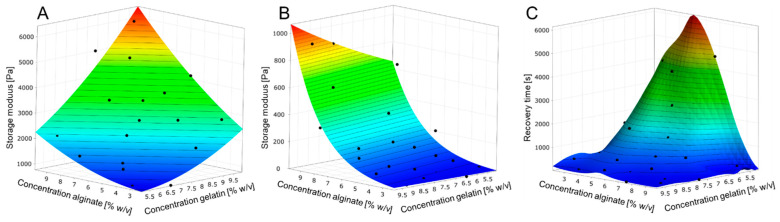
Metamodels of hydrogel behavior according to DoE. (**A**): Storage modulus depending on the gel component concentrations at 25 °C. (**B**): Storage modulus depending on the gel component concentrations at 37 °C. (**C**): Time required reaching the yield point at 25 °C after heating the hydrogel to 37 °C.

To investigate the effect of storage modulus at ambient temperature (25 °C) and 37 °C as well as the time required for a structure regeneration by cooling down from 37 °C to 25 °C, the concentration of the hydrogel components was changed. According to Figure 1, the storage modulus increases with higher concentration of each gel component and is even higher when the concentrations of both components increased. Additionally, within the viscoelastic range of hydrogels, tan δ < 1 was observed.

**Figure 2:**
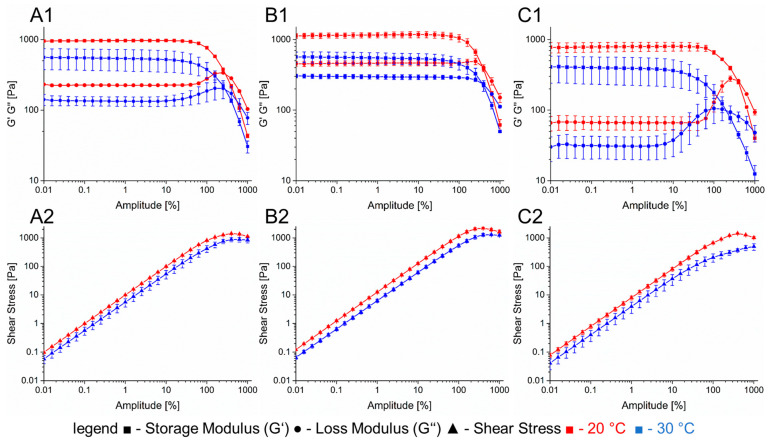
Rheological properties of the hydrogels used for printing prevascularized structures. (**A**): 6% gelatin 2% alginate. (**B**): 6% gelatin 4% alginate. (**C**): CaCl_2_-laden 7% gelatin. Section 1: complex modulus with different temperatures. Section 2: shear stress at different temperatures.

However, at 37 °C, only an increase in alginate concentration causes a higher storage modulus whereby the concentration of gelatin has no effect. Within the viscoelastic range, all samples at 37 °C exhibited tan δ > 1 showing viscous characteristics. The time to reach the yield point (tan δ = 1) after cooling down from 37 °C to 25 °C is strongly connected to the concentration of the hydrogel components. In general, the increase in one hydrogel component concentration leads to a faster gelation while this effect is intensified with higher concentrations of both gelatin and alginate. Resulting from these experiments, three hydrogels (Figure 2) were chosen for the bioprinting process and rheologically characterized at the relevant process temperatures of 20 °C and 30 °C.

**Figure 3:**
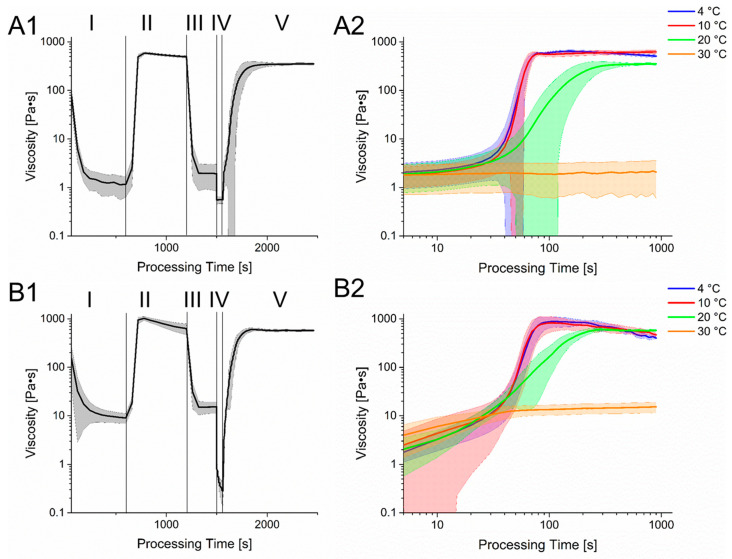
Rheological simulation of the bioprinting process. Section 1 shows the different stages of the process with varying shear rate and temperatures. Phase I: Preheating of the hydrogel for cell seeding. T = 37 °C, $\dot{\gamma }$ = 1 1/s; Phase II: Cooling of the hydrogel. T = 4 °C, $\dot{\gamma }$ = 1 1/s; Phase III: Preheat of the hydrogel for printing process. T = 30 °C, $\dot{\gamma }$ = 1 1/s; Phase IV: Bioprinting Process, T = 30 °C, $\dot{\gamma }$ = 500 1/s; Phase V: Cooling of the hydrogel on the printing substrate, T = 20 °C, $\dot{\gamma }$ = 1 1/s. Section 2 shows a more detailed course of the rheology in phase V but with different temperatures. (**A**): 6% Gelatin 2% Alginate hydrogel. (**B**): 6% Gelatin 4% Alginate hydrogel.

The first one consisted of a combination of 6% gelatin and 2% alginate that was used for printing cell-laden gels with a 250 µm nozzle. At 20 °C, this hydrogel has a storage modulus of 960 Pa that decreases to 550 Pa when heated up to 30 °C. The loss factor shows a small increase from 0.23 to 0.24 within the linear viscoelastic range. The shear force to obtain the yield point at 30 °C was measured to be 800 Pa. The second hydrogel has a combination of 6% gelatin and 4% alginate and was used as shell material for coaxial extrusion. Its storage modulus at 20 °C is 1100 Pa with a loss factor of 0.4. The temperature increase to 30 °C led to a reduced storage modulus of 560 Pa and a loss factor of 0.54. A shear force of 1200 Pa is necessary to reach the yield point. For cell laden hydrogels, the entire bioprinting process including pre-treatment and cooling was simulated with the rheometer (Figure 3).

The process begins with a heating of the gel to 37 °C. This caused a rapid decline in viscosity and enables cell seeding and homogenization. To prevent cell sedimentation and for improved process control, gel is cooled down to 4 °C, leading to an increase in viscosity again. Afterward, the gel is heated to a printing temperature of 30 °C. The printing process itself is displayed with a shear rate of 500 1/s and shows a clear reduction in viscosity for both gels, which regenerates quickly after changing the shear rate back to 1 1/s and cooling to 20 °C. However, since the used hydrogel is strongly temperature dependent, a change in the printing substrates’ temperature can alter the structure regeneration of the gel drastically.

In Figure 3, the time dependent course of viscosity after the printing process at different temperatures is displayed. While the viscosity at 30 °C returns to its pre-printing state, a quick increase can be observed at lower temperatures. However, in the beginning of the regeneration state, the increase in viscosity at 20 °C, 10 °C and 4 °C is very similar, which is connected to the temperature equilibration of the Peltier element of the rheometer. However, the viscosity at the plateau at 4 °C and 10 °C is similar and is obtained within a relative short time while the viscosity at 20 °C reduced with a longer period of time required to reach the plateau. Interestingly, the viscosity of the 6% gelatin 4% alginate hydrogel lowers after reaching its peak at 4 °C and 10 °C, leading to values less than the viscosity at 20 °C. However, since the here shown simulation of the bioprinting process was performed with a rheometer, the surface-to-volume ratio as well as temperature changes differed from the real bioprinting process. These might influence the liquidation process or gelation, especially after material extrusion.

### 3.2. Optimization of Crosslinking

The crosslinking of the 3D printed constructs was performed in a two-step process. First, CaCl_2_ was added for a fast crosslinking of the alginate part. Here, concentrations ranging from 20 mM to 100 mM were used with incubation times from 10 min to 60 min. Within the tested range, higher concentrations and incubation times showed, as listed in Table 1, a negative effect on cell viability among all three used cell lines although this effect can be considered negligible for fibroblasts. The second part of crosslinking was the addition of microbial transglutaminase with concentrations from 1 U/mL to 10 U/mL while the incubation time differed from 1 h to 24 h. As stated in Table 1, prolonged incubation times had a negative effect on cell viability while the effect of increased concentrations could be considered negligible as well. According to these results, the incubation time of CaCl_2_ was chosen to be 10 min with a concentration of 20 mM. Transglutaminase was added with a concentration of 10 U/mL and incubated for 60 min.

### 3.3. Diffusion Behavior

**Figure 4:**
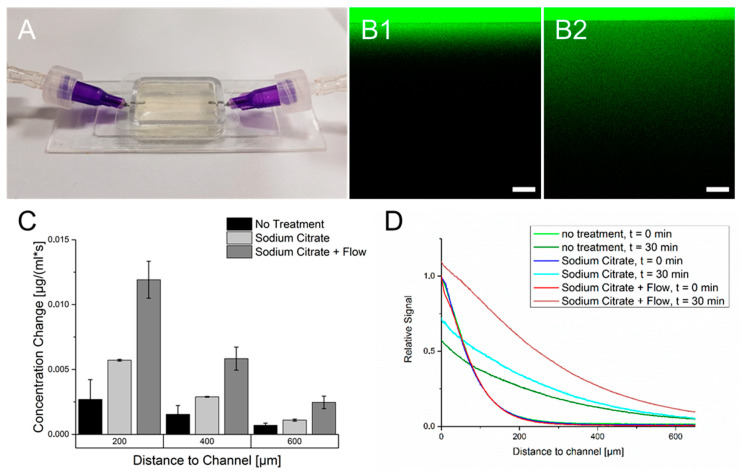
Diffusion of FITC-Dextran (20 kDa) throughout the crosslinked 6% gelatin 2% alginate hydrogel. (**A**): Mold-casted hydrogel used for diffusion analysis. (**B**): Fluorescence micrographs of FITC dextran diffusing through the hydrogel. The border between high and low fluorescence intensity marks the transition between the hollow channel and the hydrogel. (**B1**): Fluorescence directly after injection of FITC-dextran. (**B2**): FITC-dextran dispersion throughout the hydrogel after 30 min. (**C**): Calculated diffusion rates for different experiment conditions at defined distances from the supply channel. (**D**): Fluorescence signal for all three methods in relation to the channel distance and incubation time compared to the starting point at the channel wall.

The diffusion of FITC-dextran throughout the crosslinked cell-free hydrogel was investigated under three conditions. Firstly, the hydrogel was used after crosslinking without further treatment. After 30 min, due to the diffusion of FITC-dextran, an expansion of the fluorescence signal throughout the hydrogel was observed. However, the signal’s intensity decreased with prolonged distance from the supply channel, as observed in Figure 4. The second measurement was performed with hydrogels treated with sodium citrate. Here, the diffusion rate of FITC-dextran approximately doubled compared to non-treated hydrogels albeit the absolute diffusion rate of hydrogels treated with sodium citrate shows also a strong decrease with increased distance from the supply channel. The third method included the treatment of the hydrogels with sodium citrate but with a continuous addition of FITC-dextran in the channel. Here, the diffusion rate of FITC-dextran was more than twice as high as the diffusion rate with a single injection. It is also worth mentioning to consider the fluorescence signal in regions close to the channel. While FITC-dextran was added per injection, the fluorescence signal close to the channel after an incubation time of 30 min was lower than at the beginning, while the continuous addition caused a signal increase in this region. However, with regard to these measurements, one has to consider that although the image acquisition was started directly after injection of FITC-dextran, minor diffusion took place before the first micrograph. This could have led to deviations in determining the diffusion rate.

### 3.4. Effect of Treatment of Hydrogel with Sodium Citrate on Cell Viability

**Figure 5:**
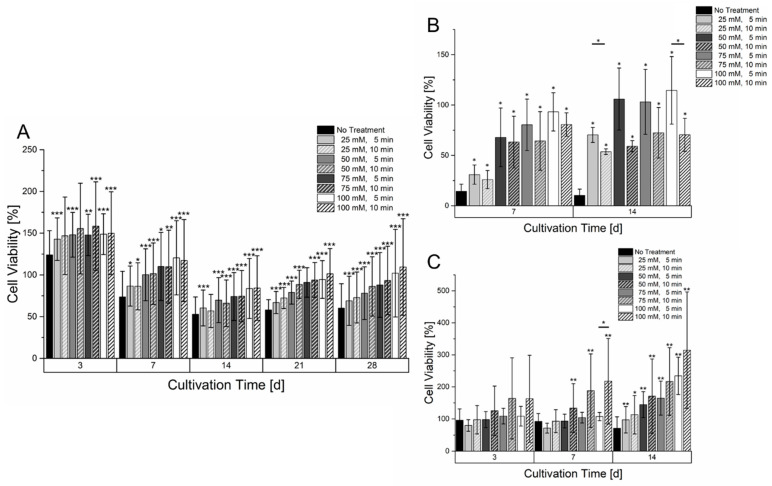
Cell viability of different mono cultures in a 6% gelatin 2% alginate hydrogel within a culture time of 14 or 28 days. (**A**): HepG2 (*n* = 4). (**B**): HUVEC-TERT2 (*n* = 3). (**C**): Fibroblasts (*n* = 4). The cell laden hydrogels were treated with different concentrations and incubation times of sodium citrate. The cell viability of each day is normalized to the signal on day 1 after bioprinting. Statistical significance was tested for all samples on each measurement day by comparing the respective sample without treatment of sodium citrate as well as with samples having the same sodium citrate concentration but different incubation time (significance level: * *p* > 0.05, ** *p* > 0.01, *** *p* > 0.005).

As could be observed in Figure 4, the hydrogels treatment with sodium citrate led to higher diffusion rates. To validate the effect on cell viability, cell-laden gels with mono-cultures of all three cell lines were printed and treated with different concentrations of sodium citrate with an incubation time of five or ten minutes. The conversion of resazurin was used as an indicator for cell viability. The time-dependent course of resazurin conversion in Figure 5 showed differences between all used cell lines. HepG2 showed an initial increase in resazurin conversion after three days of culture. Afterward, a decrease in cell viability until 14 days of cultivation could be observed. In this time, the level of resazurin conversion was less than at the start of cultivation. Afterward, a regeneration of the resazurin conversion could be observed. The treatment of the samples with sodium citrate led to an increase in cell viability. A significant increase within the entire cultivation time period could already be observed with a concentration of 25 mM sodium citrate. However, minor deviations in the significance can be caused by the limited sample size. An extended incubation time of sodium citrate seems to have a little positive effect with prolonged incubation time although no significance could be observed. Additionally, a cultivation of HepG2 in untreated hydrogels showed the formation of smaller spheroids than in gelatin-alginate hydrogels treated with 100 mM sodium citrate (Figures S2 and S3)

Cultivation of HUVEC-TERT2 cells, as shown in Figure 5, led to a different effect compared to HepG2 cells. Within the course of two weeks, HUVEC in hydrogels that were not treated with sodium citrate had a tremendous loss of cell viability that did not regenerate within the two weeks of culture. However, even samples treated with sodium citrate had a reduced resazurin conversion but showed a regeneration and partially an increase compared to a cultivation start after two weeks of culture. While sodium citrate concentrations higher than 50 mM showed similar levels of resazurin conversion after two weeks of cultivation, it was surprising to see that longer incubation times with sodium citrate led to a lower resazurin conversion. A microscopic analysis of HUVEC-TERT2 cultured in an untreated hydrogel showed that cells remained circular and isolated while those cultured in hydrogels treated with 100 mM sodium citrate formed areas of interconnected cells (Figure S5). Primary fibroblasts (Figure 5) also showed a strong dependency on the hydrogel’s treatment with sodium citrate. Low concentrations or no treatment with sodium citrate caused a stagnant and slowly decreasing resazurin conversion. With high concentrations of sodium citrate, a significant increase in resazurin conversion could be observed already after three days and was amplified with increasing cultivation times. The prolonged incubation time with sodium citrate appears to have a positive effect on cell growth within the hydrogel. However, a significant effect could only be determined after seven days of culture with a concentration of 50 mM. Here, the hydrogels treatment also showed morphological changes as with HepG2 and HUVEC-TERT2. After cultivation in untreated hydrogel, fibroblasts remained circular and isolated. When hydrogels were treated with 100 mM sodium citrate, fibroblasts adopted an elongated morphology that became more pronounced when the incubation time with sodium citrate increased (Figure S4).

### 3.5. Printing and Cultivation of Prevascularized Constructs

**Figure 6:**
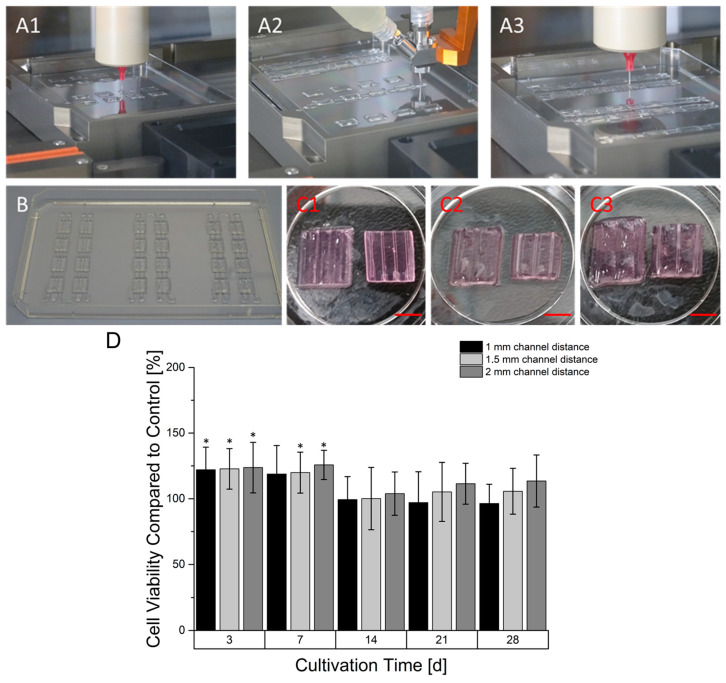
Manufacturing process of prevascularized constructs. In the first step a base layer was printed (**A1**) followed by the deposition of the coaxial printed hollow strand in a meander-linked manner over all base layer of a printing sequence (**A2**). In the last step, the space between the hollow channels was filled with another cell-laden gel (**A3**). A printing sequence created samples with one continuous hollow strand (**B**). The hollow strands that are not embedded in the hydrogel substrate were mechanically separated. The corresponding prevascularized constructs had different distances between their channels ranging from 1 mm (**C1**), 1.5 (**C2**) to 2 mm (**C3**). The left constructs still contain the core-located gelatin while the material is aspirated in the right constructs (Scale Bar: 4.5 mm). (**D**): Time dependent cell viability of prevascularized HepG2-laden constructs. The cell viability at every time point is normalized to the viability of a non-prevascularized construct serving as the control. Graphs marked with * show a statistical significance compared to avascular sample (significance level: * *p* > 0.005, *n* = 8).

Prevascularized constructs were printed in a three-step process (Figure 6), starting with printing of a cell free 6% gelatin 2% alginate basis layer for later fusion of upcoming cell-laden gels.

**Figure 7:**
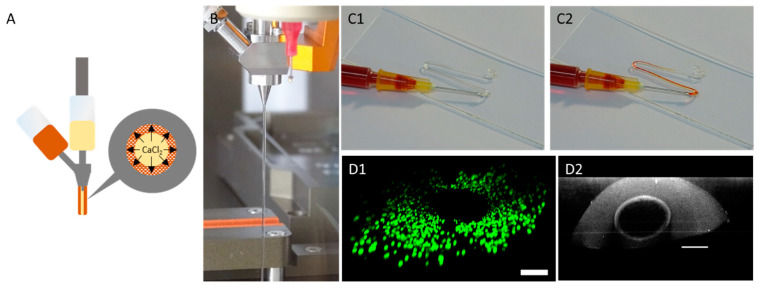
Manufacture of hollow channels using coaxial extrusion. (**A**): Principle of hollow strand formation. CaCl_2_ in gelatin (inner material, yellow) crosslinks the sheath-based gelatin (outer material, orange) causing a stable strand. (**B**): Strand formation using a coaxial extruder. (**C**): Visualization of coaxially printed strand hollowness after aspiration of core-based gelatin. (**C1**): Before addition of dye. (**C2**): After addition of dye. (**D**): CLSM micrographs showing a view through the coaxially printed strand (**D1**): View though the hollow strand with coaxially located HepG2 cells. Visualization of the cells is achieved using live–dead staining. Green: live cells; red: dead cells. (**D2**) Visualization of the hollow strand using reflection microscopy using a wavelength of 405 nm (scale bar: 200 µm).

During a printing process, different design versions of prevascularized constructs were printed. For every version, multiple base layers were fabricated. In the next step, using coaxial extrusion, a core/shell designed strand was continuously printed over the base layer in a meander like manner. The core-section of the printed strand consisted of a 7% gelatin supplemented with 100 mM CaCl_2_ causing an instantaneous crosslinking of the sheath-located cell-laden 6% gelatin 4% alginate hydrogel. For every design version, different distances between the were channels created, namely 1 mm, 1.5 mm and 2 mm. In the last step, the gaps between the coaxial printed strands were filled up by printing another cell laden 6% gelatin 2% alginate hydrogel. The generation of the hollow strand was completed after alginate crosslinking with CaCl_2_ in the incubator. During this time, the core-located gelatin in the coaxially printed strand liquified and was able to be aspirated. As shown in Figure 7, the strand could be perfused. In addition, cells could be located around the core although it was not perfectly homogenized. The cultivation of prevascularized constructs was found to be superior in terms of resazurin conversion compared to avascular constructs in the first seven days of culture, as observed in Figure 6. However, after 14 days of culture, the resazurin conversion of prevascularized cultures had an approximate level similar to the avascular samples. With longer cultivation, prevascularized samples with a channel distance of 2 mm appeared to gain an increasing level of resazurin conversion.

### 3.6. Continuous Cultivation of Prevascularized Constructs

**Figure 8:**
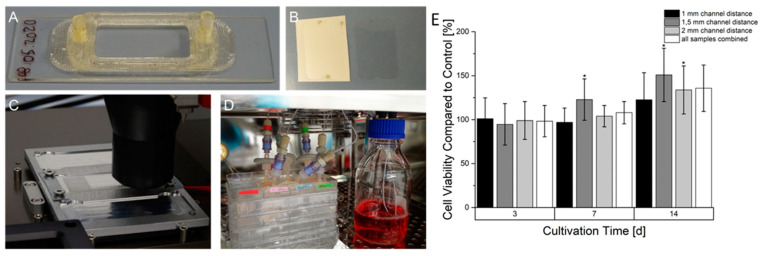
Manufacturing process, set up and resazurin conversion of HepG2 in prevascularized constructs after continuous cultivation. (**A**): 3D printed miniaturized bioreactor made of COC printed on a COC slide. (**B**): Laser structured adhesive foil on carrier foil (left) and after removal from carrier foil (right). (**C**): Printing process of the bioreactor on a heated aluminum-based slide carrier. (**D**): Set-up of bioreactors in the incubator connected to the fluidic system. (**E**): Time-dependent cell viability of continuously cultured prevascularized HepG2-laden constructs. The cell viability at every time point is normalized to an equivalent statically cultured construct serving as a control. Graphs marked with * show a statistical significance compared to their statically cultured equivalent (significance level: * *p* > 0.005 *n* = 6).

The 3D printing of COC 5013 using the high temperature extruder achieved a good adhesion of the molten plastic on the pre-heated COC substrate. The following layers also showed good adhesion causing a leak-proof reactor. However, a major problem of 3D printed parts based on extrusion printing is waviness, which might prevent the connection of the adhesive foil. However, due to parameter optimization, the reactors surface around the chamber showed a relative smooth surface that enabled the adhesion of the sealing foil. The 3D-printed miniaturized bioreactor itself, as observed in Figure 8, consisted of two tube plugs connected to an open chamber reserved for insertion of 3D printed samples. This allows, depending on the size, the insertion of multiple samples. The performance of the resazurin assay (Figure 8) showed in comparison with static cultivated prevascularized samples an almost identical signal within seven days of cultivation. However, after 14 days of continuous cultivation a higher signal in samples with continuous cultivation could be observed. Among all samples in the bioreactor, significant differences could be observed after 14 days. Overall, 1.5 mm samples showed the highest signal while 1 mm samples had the lowest signal.

### 3.7. Co-Culture of Prevascularized Constructs

**Figure 9:**
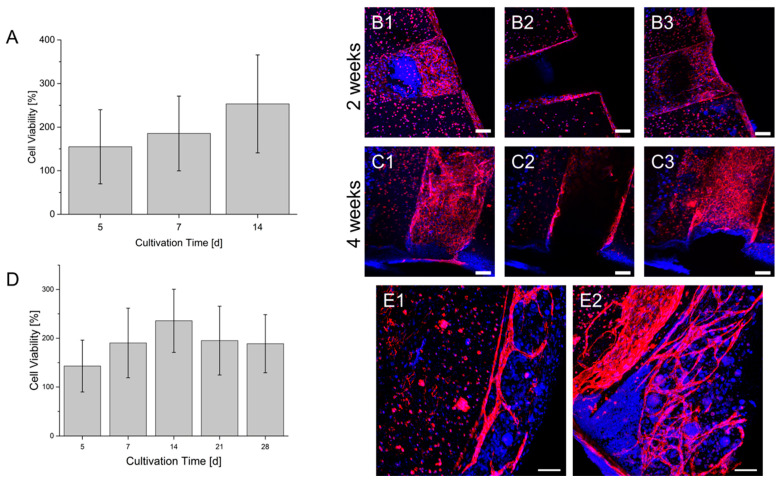
Co-Cultures of HUVEC and HepG2 (**A**–**C**) and HUVEC, HepG2 and fibroblasts (**D**,**E**) in a prevascularized construct. (**A**): Cell viability of the co-culture within a culture time of 2 weeks. (**B**): Endothelialization of the hollow strand by HUVEC in the co-culture after 2 weeks. (**C**): Endothelialization of the hollow strand by HUVEC in the co-culture after 4 weeks. Sections 1–3: Maximum intensity projection of different segments of the hollow strands. 1: lower area; 2: middle area; 3: upper area. A scheme of the visualized areas can be seen in the Supplementary Materials. (**D**): Cell viability of the triplet co-culture within a culture time of 4 weeks. The cell viability of each day is normalized to the signal on day 1 after bioprinting (also in A). (**E1**): Formation of vascular structures by HUVEC in the co-culture after 2 weeks. (**E2**): Formation of vascular structures by HUVEC in the co-culture after 4 weeks. Blue: DAPI; Red: CD 31; Scale bar: 100 µm.

In a first model, HUVECs were located in the shell of the hollow strands while HepG2 cells were printed between the hollow strands. Resazurin conversion (Figure 9) showed within two weeks of culture a continuous increase in the signal. However, the immunofluorescence of HUVEC by CD31 staining showed a population of the hollow channels surface that is indicated after two weeks of culture and is enhanced after four weeks. However, in addition to the population of hollow channels, HUVEC remained in a circular shape, showing no tendency for migration or proliferation. In another co-culture, HepG2 cells and primary fibroblasts were mixed in one gel and printed between the hollow channels containing HUVEC. Similarly to the co-culture without fibroblasts, the resazurin conversion increased until 14 days of culture (Figure 9). However, afterward, a decline in conversion could be observed. The major difference between the cultures without fibroblasts is the growth of HUVEC. Here, HUVEC show a sprouting behavior with a directed growth toward HepG2 and fibroblasts. The growth of typical vascular structures could be observed after 14 days of culture and was even enhanced after 28 days of culture.

## 4. Discussion

### 4.1. Hydrogel Characterization

The rheological behavior of hydrogels is one of the main factors determining printability. A major feature of gelatin is its thermo-reversible gelation behavior due the known helix-coil transition at higher temperatures leading to a liquidation [23]. Alginate shows also a temperature dependency causing lower viscosity [24], although this effect is much less drastic than for gelatin. The used temperatures of 25 °C and 37 °C for the characterization of different gelatin-alginate hydrogels present the state of the hydrogel before the bioprinting process and after heating for cell seeding and also two different conditions of the hydrogel. A temperature dependent loss of the storage modulus could be observed in the performed DoE by increasing the temperature from 25 °C to 37 °C. The main reason of this is the mentioned liquidation of gelatin. However, this loss could also be amplified due to the hydrogel preparation that was performed in a steam cooker. It is known that higher processing temperatures lead to reduced mechanical stability [16]. The treatment in the steam cooker might have enhanced the liquidation process of gelatin at 37 °C so that it did not show any influence in the storage modulus. However, the high temperature treatment could also have influenced the mechanical stability of alginate, leading into reduced storage modulus too. This thermal behavior of the hydrogel blend offered various strategies for adjusting the bioprinting process. In the pre-bioprinting-process, gel was heated to allow cell seeding. However, as shown by a study of Dubbin et al. [25], cell sedimentation occurs in low viscous hydrogels causing an inhomogeneous cell distribution within the hydrogel. To prevent this, a short-term storage of cell-laden hydrogels at 4 °C after the seeding process was performed. Afterward, the gels were transferred into the bioprinters cartridge heater to adjust them to a printing temperature of 30 °C that showed a rapid and consistent adjustment of viscosity. Another approach for the pre-bioprinting-process was performed by Jiang et al. [26]. In their study, the hydrogel was cooled down to 25 °C after previously heating to 32 °C and cell seeding. After 50 min of cooling, the time slot for printing was achieved. However, during this printing time the viscosity showed a continuous increase that might alter the flow rate when printing with continuous pressure. Instead, heating from a chilled hydrogel allows controlled energy inputs and a constant viscosity after a shorter amount of time, as shown in Figure 3. Another approach for the preparation of the bioprinting process was proposed by Ouyang et al. [27]. Here, they started with a temperature of 37 °C for the preparation of the bioink and cooled the cartridge to a temperature between 22.5 °C to 30 °C for investigating the printing process while the graft was printed into an isolated chamber with a temperature of 22.5 °C. Using time-sweep tests, they showed that the complex modulus increased faster with lower temperature, also resulting in a higher storage modulus. However, even after extensive measurement times, an ongoing increase in complex modulus was observed that could alter the printing process negatively by altering the size of the fabricated structure depending on the timepoint of fabrication.

The selected hydrogel blends consisting of 6% gelatin 2% alginate as well as 6% gelatin 4% alginate hydrogel showed low viscosities at a printing temperature of 30 °C. This allowed printing relatively low air pressures and preserves cell viability. Gao et al. [28] performed a printability analysis with gelatin-alginate hydrogels by measuring the complex modulus. There, they proposed a loss factor ranging from 0.25 < tanδ < 0.45 to achieve smooth three-dimensional structures. While the 6% gelatin 2% alginate has a loss factor that is almost within this range, the 6% gelatin 4% alginate hydrogel deviates from this range at printing temperatures. However, using these hydrogels with their corresponding tools, functional printing to create full thickness constructs was achieved. However, since hydrogels with low viscosity are vulnerable for structure loss, a fast gelation process after the bioprinting process needs to occur. Similarly to that mentioned by Paxton et al. [27], post-bioprinting temperatures can be altered to regulate rheological behavior of the bioprinted hydrogels. While the hydrogels show a rapid regeneration of their pre-printing viscosity, the cooling procedure caused an increase in viscosity depending on the used temperature. Interestingly, the rheological investigation showed an almost identical course of viscosity increase and plateau formation at 4 °C and 10 °C. Since single-printed struts show a high surface to volume ratio, this thermal-based gelation process should be reproducible in a real bioprinting process, providing the constructs with relatively high mechanical stability. Using a low temperature substrate, multi-layered porous structures should be printable. However, for printing full-thickness constructs, a temperature of 20 °C was used. Here, the gelation process required more time, providing the possibility for the printed structures to fuse. Eventually, mechanical stability also increases, providing the printed constructs with sufficient stability.

### 4.2. Effects on Crosslinking

Crosslinking was performed in a dual-stage process beginning with ionic crosslinking of alginate followed by enzymatic crosslinking of gelatin. Ionic crosslinking of alginate using Ca^2+^ as bi-cation originated from CaCl_2_, caused the linking of guluronic acid side groups [29]. Although CaCl_2_ does not show any toxic effects on cells, increased incubation time and concentration led to reduced resazurin conversion. This could be caused due to the advanced complexity of the alginate network limiting diffusion and cell motility inside the hydrogel. Especially since alginate shows a bio-inert behavior, advanced crosslinking of alginate might reduce cellular functionality. In a study of Gudapati et al. [30], a negative effect of prolonged CaCl_2_-incubation was also described. This caused the formation of a thicker alginate membrane, reducing the diffusion of glucose and oxygen and resulting in reduced cell viability. Since alginate crosslinking is required to achieve the mechanical stability of the graft at 37 °C prior to transglutaminase crosslinking, a low concentration of 20 mM with a short incubation time of 10 min of CaCl_2_ was used. Regarding the crosslinking of gelatin with microbial transglutaminase, an increased concentration improved cell viability while prolonged incubation time had a contrary effect. Transglutaminase forms iso-peptide bonding based on an acyl-transfer using carboxyl-residues from glutamic acid and amino residues from lysine. During this reaction, ammonia was formed [31], which is known to have a harmful effect on cells [32]. Therefore, the incubation time of one hour was chosen to minimize the contact of cells with ammonia and to minimize harmful effects. However, the effect of transglutaminase concentration appeared to be very small, which could indicate that the degree of gelatins crosslinking was sufficient at all concentrations used. However, a higher concentration of transglutaminase was used to increase mechanical stability of the cell-laden hydrogel, which is why a concentration of 10 U/mL was used for later experiments. In addition to the effect on cell viability, crosslinking will also affect the mechanical stability of the hydrogel. CaCl_2_ was added first to the hydrogel to achieve a temperature stable hydrogel for the cultivation of the cell laden grafts at physiological temperatures in the incubator. A prolonged incubation time and higher concentrations of CaCl_2_ would have led to higher mechanical stability. This effect has been found by Ramdhan et al. [33] that varied the mentioned parameters in the formation of alginate cuboids. However, they also saw that, after a certain incubation time that depended on the used concentration, stability remained constant or was slightly decreased due to rearrangement of the ions in the network. The used parameters for alginate crosslinking might have an influence on the gelatin crosslinking with transglutaminase. A high-density network of crosslinked alginate will probably hinder the diffusion of transglutaminase, causing an uneven crosslinking of gelatin. Therefore, higher incubation times are necessary to achieve a complete crosslinking of gelatin. However, the mechanical stability of gelatin crosslinked with transglutaminase depends also on the used concentration and incubation time. Zhou et al. [34] investigated the gelation kinetics of GelMA at 37 °C by assessing viscosity. They found an increase in gelation rate with increasing concentrations. With their highest concentration and incubation time used, the viscosity of GelMA appears to shift into a plateau state. A more extensive characterization of gelatin crosslinked with transglutaminase has been performed by Cacopardo and Ahluwalia [35]. Here, they compared the properties of gelatin gels that were crosslinked once for a relative short time with other specimen that underwent an additional long-term crosslinking step to assess the influence of prolonged transglutaminase incubation. They determined that prolonged crosslinking with transglutaminase causes resistance to hydrolytic degradation but also continuously decreases the hydrogels’ water storage capability. Moreover, they also observed a higher stiffness with increasing incubation time as well as a lower cell viability compared to shorter incubation times. The effect of reduced cell viability coincides with the findings of our work that supports the choice of a shorter incubation time for crosslinking with transglutaminase.

### 4.3. Diffusion Behavior of Crosslinked Hydrogels

The diffusion of FITC-dextran with a molecular weight of 20 kDa was performed to simulate the movement of VEGF inside the cell-free crosslinked hydrogel. Similarly to native tissue, the diffusion range of FITC-dextran showed a rapid decrease with increasing distance from the nutrient channel. As stated by Wu et al., this behavior can be explained by various physical models, including a smaller free volume of hydrogels or longer pathways caused by additional obstructions [36]. To improve the diffusional behavior, sodium citrate was added, which is known to remove the ionic crosslinking of alginate [37]. Citrate acts as a chelator binding calcium ions from crosslinked guluronic acid side groups, causing alginates liquidation. Due to higher porosity and less obstructions inside the hydrogel, diffusion improved. The highest rate of diffusion was observed when adding FITC-dextran continuously in the channel. Due to the constant addition of material, a high concentration gradient could be maintained. This caused a faster diffusion of FITC-dextran into regions that are further away from the channel, leading to a higher signal compared to experiments with a single injection of FITC-dextran. Due to the continuous mass transfer of the chemical into the hydrogel, FITC-dextran was enriched in the hydrogel. This could particularly be detected in approximate regions of the supply channel. Here, the fluorescence signal increased 30 min after continuous addition of FITC-dextran. In contrast, the signal in this region decreased after the single material injection. Consequently, a perfused 3D culture will obtain the best media supply but requires a prevascularization in order to maintain short diffusion paths. Another extensive study of diffusional behavior was performed by Nie et al. [38], who used a GelMA hydrogel and two concentrations of FITC-dextran. They showed that FITC-dextran with a lower molecular weight had a longer diffusion path within the same time interval. This could indicate that diffusion of small molecules such as glucose will penetrate more deeply into the hydrogel while cell signaling with larger molecules is limited to smaller areas. Additionally they determined that a monolayer of HUVEC on the inside of the channel caused a reduction in FITC-dextran diffusion throughout the hydrogel, which was also observed by Kolesky et al. [16]. Alhough endothelial cells form a natural barrier in the endothelium, it also indicates that the cells surrounding the supply channel might hinder the diffusion of molecules throughout the hydrogel.

### 4.4. Mono Cultures of Cell Lines in Avascular 3D Constructs

All three cell lines tested showed a different time-dependent behavior regarding their resazurin conversion. In general, all cell lines benefited from alginate removal due to treatment with citric acid. A major beneficial factor is the mentioned improved diffusion throughout the hydrogel. Yet another factor could be the altered mechanical stability of the hydrogel after the removal of alginates crosslinking (Figure S1). With regard to HepG2 cells, higher concentrations of citric acid and potentially a prolonged incubation time improved cell viability and motility. However, after a short term increase in viability, a mid-term decrease could be observed followed by long-term regeneration. HepG2s were originated from a hepatocellular carcinoma originating from the liver that facilitates high-energy consumption [39]. In conjunction with the relatively high cell concentration and the pore-free design of the construct, nutrient supplies could be limited throughout the hydrogel, causing necrosis. Since the signal of the used resazurin assay correlates with the number of viable cells, it appears that regrowth occurs, which could also be seen in live–dead staining. In a study of Kang et al. [40], HepG2 showed a different viability according to their distance in the lumen. While cells remained viable close to the surface or a lumen, HepG2 in between showed decreased viability. In conclusion, it is possible that the regrowth of HepG2 in avascular constructs took place primarily in the outer regions. Sodium citrate treatment of the fibroblast laden hydrogel construct showed similar effects as for HepG2 cells. Fibroblasts showed an elongated growth and sprouting on treated hydrogels while they retained a circular shape in untreated hydrogels. The same effect was observed by Gilette et al. [41] in an alginate–collagen hydrogel when ionic crosslinking of alginate was dissolved using citric acid. The influence of alginate concentration in a gelatin-alginate-dialdehyde was investigated by Sarker et al. [42]. They showed that a higher ratio towards gelatin while reducing the concentration of alginate-dialdehyde improved proliferation in this gel. In terms of alginate removal with sodium citrate, the ratio of the hydrogel components is also shifted toward gelatin, which might support fibroblast growth. Furthermore, in another study, they determined the positive effect of alginate–dialdehyde crosslinking with gelatin. This caused a porous structure of the hydrogel, providing fibroblasts with a better possibility for sprouting and proliferation [43]. However, with appropriate citrate treatment, resazurin conversion showed a continuous signal increase over two weeks of culture and differs strongly from HepG2 cells. This might be connected to the reduced cell concentration causing an improved availability of nutrients. The growth of HUVEC cells in sodium citrate treated hydrogels differs from the growth of HepG2 cells and fibroblasts. Increasing concentrations until 50 mM of sodium citrate led to improved cell proliferation, yet prolonged incubation time led to decreased viability. Sodium citrate has the possibility to reduce cell viability in cancerous cells due to acidification [44]. However, in this study cells were exposed to sodium citrate for at least 24 h while the maximum incubation time for HUVEC cells was 10 min. Another reason might be the different requirements for the microenvironment of HUVEC cells compared to fibroblasts and HepG2 cells. This was also stated by Liu et al. [45] who investigated the growth of different cell lines in GelMA-hydrogels with different stiffness. Thereby, the optimal material for growth of HUVEC differed from the other cell lines. After the proper preparation of the hydrogel by crosslinking both hydrogel components with the subsequent removal of alginate, HUVEC showed an increase in resazurin conversion after two weeks. In another study by Benning et al. [46], a gelatin–alginate hydrogel film was prepared without the crosslinking of gelatin. Here, the growth of HUVEC was not functional, which might show the necessity to crosslink the gelatin in this hydrogel.

### 4.5. Fabrication and Cultivation of Prevascularized Constructs

The fabrication of prevascularized constructs was performed in a three-step process starting with the deposition of an acellular 6% gelatin 2% alginate hydrogel that served as a substrate for the latter printed structures. The second step included the printing of a pre-form of hollow channels using coaxial extrusion. The used coaxial extrusion tool consisted of a core and a shell compartment, which is the most widespread configuration. However, Pi et al. used a coaxial extruder consisting of three inlets, providing the possibility to create multilayered hollow channels [47]. Cao et al. used the same coaxial extrusion set-up to crosslink an alginate hydrogel from inside as well as outside [48]. This double-sided crosslinking procedure shows beneficial possibilities for creating single hollow tubes since every site of the strand is crosslinked. Instead, the hollow channels created in this work were crosslinked from the center, which might lead to a non-crosslinked segment in the outer part due to insufficient diffusion. However, for the creation of prevascularized full thickness structures, this outer part can be used for the fusion of the subsequently printed structures. Nevertheless, the printed structures in this study are additionally crosslinked directly after printing by the thermal gelation of gelatin due to the temperature of the well plate holder. The addition of another cell laden gel in between the hollow strands allows the creation of full thickness constructs. Despite the observed negative effect of alginate crosslinking with a high concentration of CaCl_2_, a 100 mM solution was used to achieve proper fusion. Since the hydrogels were treated with sodium citrate, the negative effects might be neglected. The use of gelatin as a carrier of CaCl_2_ for crosslinking the sheath-located gelatin-alginate hydrogel is beneficial since alginate crosslinking takes place at 37 °C in the incubator, leading to the liquidation of gelatin. This allows an aspiration of the core-located material, enabling the generation of hollow channels. Another popular sacrificial material is Pluronic F127, which liquefies at lower temperatures [49]. However, this would require a placement, e.g., in the refrigerator, causing an additional process step. The improvement in cell viability using prevascularized constructs compared to avascular constructs has also been shown by other groups. Bertanossi et al. used MC3T3 cells encapsulated in a GelMA hydrogel that was either formed as a block or a block with a hollow channel. Cell viability remained higher in the vascular block compared to the avascular variant within the measured time range of seven days [50]. Gao et al. also observed improved cell viability in constructs with microchannels compared to hydrogel constructs without hollow tubes. Here, L929 mouse fibroblasts were cultured in alginate gels lacking RGD-motifs [19]. The missing adhesion ligands in the hydrogel might be the reason for the continuous decrease in cell viability within the tested time range. However, the observed analysis of cell viability was performed for seven days. In this study, the beneficial effect of prevascularization was observed too, but it declined after prolonged culture time. After 14 days of culture, the prevascularized cultures showed the same level of resazurin conversion similarly to the avascular samples. This might be connected to the increased cell number after short term culture, which could be sufficiently supported with nutrients due to shortened diffusion paths. A reduced diffusion speed due to increased cell numbers has been shown by Cao et al. [48]. Due to this increase in cells in the construct, more consumer of nutrients and obstacles hinder the diffusion leading to gradients. This might lead to areas that were not adequately supported with nutrients, leading to necrosis. The existence of such areas has been shown by the previously mentioned study of Kang et al. [40]. In long-term cultures, the resazurin conversion of prevascularized structures showed a small increase in the level of resazurin conversion compared to the avascular level. Here, the highest level of resazurin conversion appears for samples with a channel distance of 2 mm. However, the reason for this needs to be further investigated.

### 4.6. Continuous Cultivation of Prevascularized Constructs

The manufacture of a miniaturized bioreactor using the plastic COC 5013 offers many advantages such as mechanical stability, low protein adsorption, a chemical resistance against aqueous solutions or thermal stability, enabling the action of autoclaving the reactor. The design allows an easy transfer of prevascularized constructs with subsequent sealing with an adhesive foil. A drawback of this system that was later found is a lack of gas permeability. The only gas inlet is caused by the buffered culture medium for which its storage bottle is located in the incubator as well. Another bioreactor was additively fabricated by Kolesky et al. [16] using silicone to produce a shell for the subsequently printed cell laden hydrogel. The prevascularized network was directly connected to the fluidic system, allowing direct perfusion through the hollow channels. Sealing of the chip was achieved by placing an acrylic lid on the top of the chip. Another approach was performed by Lee et al. [51] who additively manufactured a chip made of polycaprolactone and printed cell-laden hydrogels into this shell with different designs. They showed that a perfusion based culture of hepatocytes led to an increased formation of albumin and urea compared to static cultivation. The continuous cultivation of the prevascularized samples in the bioreactor might show two effects. First, up to seven days, the resazurin signal between static and continuously cultivated samples remained similar, which might show that the nutrient supply in static cultivated samples is sufficient. Another reason might be the gas supply in bioreactors. All components for fabrication and sealing were made with polyolefins that have low gas permeability. Consequently, the only gas inlet is achieved by media influx, for which its supply vessel is also located in the incubator. This reduced amount of gas inlet could also lead to reduced growth of cells in the gel. However, after 14 days of cultivation, the continuously cultivated samples showed a significant higher signal than constructs cultivated with static conditions. At this point, the nutrient support on continuously cultivated samples might became superior due to limited diffusion in the statically cultivated constructs. The second effect seen in this cultivation is the position of the constructs inside the bioreactor. For these experiments, two samples of each design were transferred into the chip und cultivated. However, the samples of 1 mm distance were placed more closely to the outlet while the 1.5 mm design was positioned primarily close to the inlet. The used flowrate of 25 µL/min caused a laminar flow inside the chip, causing diffusion-based nutrient exchanges. Due to the excess amount of nutrients in the reactor inlet, samples located to this position are better supplied than at the outlet, causing a higher cell viability.

### 4.7. Co-Culture of Prevascularized 3D-Cultures

Co-Culture of HUVEC with HepG2 cells showed a progressively endothelialization of the hollow channel within four weeks of culture. However, HUVEC around the hollow channel appear quiescent and remain in a circular shape. Although HepG2 express VEGF in culture, forming a VEGF-gradient towards HUVEC, no sprouting could be observed. However, this co-culture already showed the potential of coaxial bioprinting to form hollow strands with on-site delivery of HUVEC that can later form functional structures. Furthermore, with the co-culture of fibroblasts and HepG2, sprouting could be observed. HepG2 are epidermal cells originated from a hepatocellular carcinoma facilitating metabolic activities but are not directly involved in angiogenesis processes. Instead, fibroblasts are an essential cell line in the structure of blood vessels, producing the surrounding extracellular matrix and expressing proangiogenic cytokines. Although HUVEC and fibroblasts were spatially divided in the prevascularized constructs, the sprouting of HUVEC occurred. In the micrographs, a directed growth of HUVEC toward the compartment with HepG2 cells and fibroblasts can be anticipated. However, the expression of collagen I inside the cell-laden hydrogel was proven, as well as the expression of vWF (Figures S6 and S7).

Another endothelialization of a hollow channels has been made by Thomas et al. [52]. Using stereolithography, they created a preform of a vascularized constructs made of GelMA, using a HUVEC-laden sacrificial hyaluronic acid-based bioink. After 28 days of culture, HUVEC formed an endothelialized channel. However, since this construct consisted of HUVEC only, no further growth out of the channel could possibly be observed. Zhu et al. [53] performed another stereolithography-based approach. In this study, HUVEC and fibroblasts were printed into the surroundings of a honeycomb-like structure that remained hollow, forming prevascularized structures. Cells were embedded in a blend of methacrylated gelatin and hyaluronic acid and formed an endothelial network after one week of culture. These results as well as our findings suggest that gelatin-based materials can be used for the formation of endothelial structures. Hong et al. [54] used coaxial extrusion to print hollow cell laden struts with HUVEC and fibroblasts. They used gelatin modified with PEG and tyramine loaded with HRP as the outer shell, while a combination of gelatin, and H_2_O_2_ was located in the shell. A fibrin gel surrounded these strands. After 8 days, the formation of an endothelial cell layer inside the vascular channel could be observed. In agreement with these results, coaxial printing allows the formation of hollow strands by delivering endothelial cells on-site during the bioprinting process. Additionally in this study, the outgrowth of HUVEC from the coaxial printed compartment toward adjacent cell-laden hydrogels has been shown. However, a drawback of this technique compared to printing a single sacrificial material is the lack of printing a layer that is wisely connected vascular network. When using a single sacrificial material, junctures for the hollow vessels can be created layer-wise with a high degree of freedom. Instead, since coaxial extrusion produces a core that is always surrounded by a shell, continuous extrusion is necessary in order to form a connected network. To create a multilayer connected vascular network, it is necessary to continue extrusion when switching to the next layer. This reduces the possibility of designs, since either the coaxial printed strands will be stacked or, when overhanging structures are created, no material under these can be printed. However, printing layer-independent networks is possible and these might connect each other to vascular networks by angiogenesis processes. Again, a major advantage is the on-site delivery of cells to the hollow strands, dismissing the necessity for a post-seeding procedure after the aspiration of the sacrificial material.

## 5. Conclusions

In this study, the fabrication of prevascularized full-thickness constructs using coaxial extrusion to produce hollow channels was established using different hydrogels based on a gelatin and gelatin-alginate-blends. The characterization of the hydrogel components and the used hydrogels thermal behavior in terms of its rheological properties was investigated toward a workflow-focused approach that could be adapted for other temperature-dependent gelatin-based bioinks. In particular, the possibility for adjusting the gelation speed of the used gelatin-alginate hydrogels using different substrate temperatures was shown. The dual-stage crosslinking procedure was optimized to enhance cell viability, showing that alginate crosslinking should be performed with low concentrations and short incubation times with CaCl_2_, while transglutaminase used for gelatin crosslinking should be added with high concentrations but shorter exposure times. In addition, the importance of alginate removal using sodium citrate to improve cell growth has also been shown for cultivation with HepG2, fibroblasts and HUVEC, causing an increase in cell viability as well as an alteration of cellular morphology after citrate treatment during prolonged cultivation times. The impact of alginate removal could also be shown with respect to reduced mechanical stiffness according to rheological investigations as well as in an increased diffusion of FITC-dextran within hydrogels treated with sodium citrate. The manufacture of prevascularized constructs was possible using coaxial as well as single extrusion tools in a three-step process. The hollow channels were surrounded by avascular parts for which their distance to each other can easily be altered. Due to its flexibility and the possibility to insert different cell laden materials between the channels, it is very well suited for biological interactions between different spatially divided cell lines in a 3D culture system. Long-term cultivation of these prevascularized constructs using a monoculture HepG2 showed a short-term beneficial effect that was negated in mid-term cultivation. Using a perfused culture, cell viability could be improved at mid-term cultivation times. This was performed by using a resealable 3D-printed miniaturized bioreactor made of plastic COC 5013 that allowed the insertion of bioprinted samples and easy installation. The developed prevascularized constructs were further used for studies of co-cultures with HepG2, fibroblasts and HUVEC, while HUVEC were always localized around the hollow channels. It was found that co-cultures of different cell lines led to improved resazurin conversion compared to single culture. The spatial placement of HUVEC around the hollow channel led to its endothelialization when co-cultured with HepG2, but no sprouting could be observed. With the addition of fibroblasts, sprouting occurred. These results show that coaxial extrusion offers a great possibility for the on-site delivery of HUVEC to a hollow channel offering a good starting point for endothelialization and vascularization. In addition, the developed prevascularized constructs offer a good base for specific tissue models such as liver by substituting HUVEC with liver sinusoidal cells. In conclusion, this development shows that prevascularization of pore-free co-cultures on the example of HepG2, fibroblasts and HUVEC can lead to enhanced growth and better survival compared to non-prevascularized mono-cell cultures, providing an important step in the development of advanced three-dimensional cell cultures models.

## Figures and Tables

**Table 1:** Pearson’s coefficient showing the influence of crosslinkers process parameter on cell viability. A value greater than zero indicates a positive effect on cell viability and vice versa. The range for the indicator.

Cell Line	Concentration CaCl_2_	Incubation Time CaCl_2_	Concentration mTGM	Incubation Time mTGM
HepG2	−0.59	−0.23	0.13	−0.34
HUVEC-TERT2	−0.34	−0.47	0.06	−0.07
Fibroblasts	−0.13	−0.05	−0.04	−0.53

## Data Availability

Not applicable.

## Supplementary Materials

The following supporting information can be downloaded at: https://www.mdpi.com/article/10.3390/bioengineering9060242/s1, Figure S1: Complex modulus of 6% gelatin 2% alginate hydrogel at different phases during crosslinking. A: 10 min treatment with 20 mM CaCl_2_. B: 60 min treatment with 10 U/mL transglutaminase after CaCl_2_ treatment. C: 5 min treatment with 100 mM sodium citrate post-crosslinking, Figure S2: Live–Dead Staining of HepG2 in 6% gelatin 2% alginate hydrogels treated with sodium citrate at various timepoints during cultivation. Green: live cells (calcein AM). Red: dead cells (propidium iodide). Scale Bar. 100 µm, Figure S3: Live–Dead Staining of HepG2 in a 6% gelatin 2% alginate hydrogels without treatment of sodium citrate at various timepoints during cultivation. Green: live cells (calcein AM). Red: dead cells (propidium iodide). Scale Bar. 100 µm, Figure S4: Live–dead Staining of fibroblasts in 6% gelatin 2% alginate hydrogels after 14 days of culture in gels with different post-crosslinking treatment. A: No treatment with sodium citrate. B: 5 min treatment with 100 mM sodium citrate. C: 10 min treatment with 100 mM sodium citrate. Green: live cells (calcein AM). Red: dead cells (propidium iodide). Scale Bar. 100 µm, Figure S5: Immunfluorescence staining of HUVEC in a 6% gelatin 2% alginate hydrogel after cultivation for two weeks with different post-crosslinking treatment. A: No treatment with sodium citrate. B: 5 min treatment with 100 mM sodium citrate. Blue: Nucleus (DAPI). Red: CD31. Scale Bar: 100 µm, Figure S6: Immunstaining of van Willebrandt Factor of HUVEC. Blue: Nucleus (DAPI). Red: vWF. Scale Bar: 100 µm, Figure S7: Immunfluorescence Staining of collagen I in a co-culture of HepG2, fibroblasts and HUVEC in prevascularized constructs after 4 weeks. Blue: Nucleus (DAPI). Red: collagen I. Scale Bar: 100 µm, Figure S8: Scheme showing the principal design of the prevascularized construct with the areas that are displayed in Figure 9B,C.

## References

[1] Thouand (2020). Handbook of Cell Biosensors.

[2] Hofmann U., Michaelis S., Winckler T., Wegener J., Feller K.-H. (2013). A whole-cell biosensor as in vitro alternative to skin irritation tests. Biosens. Bioelectron..

[3] Hofmann U., Priem M., Bartzsch C., Winckler T., Feller K.-H. (2014). A sensitive sensor cell line for the detection of oxidative stress responses in cultured human keratinocytes. Sensors.

[4] Saydé T., El Hamoui O., Alies B., Gaudin K., Lespes G., Battu S. (2021). Biomaterials for Three-Dimensional Cell Culture: From Applications in Oncology to Nanotechnology. Nanomaterials.

[5] Dubiak-Szepietowska M., Karczmarczyk A., Jönsson-Niedziółka M., Winckler T., Feller K.-H. (2016). Development of complex-shaped liver multicellular spheroids as a human-based model for nanoparticle toxicity assessment in vitro. Toxicol. Appl. Pharmacol..

[6] Dubiak-Szepietowska M., Karczmarczyk A., Winckler T., Feller K.-H. (2016). A cell-based biosensor for nanomaterials cytotoxicity assessment in three dimensional cell culture. Toxicology.

[7] Carmeliet P., Jain R.K. (2000). Angiogenesis in cancer and other diseases. Nature.

[8] Unagolla J.M., Jayasuriya A.C. (2020). Hydrogel-based 3D bioprinting: A comprehensive review on cell-laden hydrogels, bioink formulations, and future perspectives. Appl. Mater. Today.

[9] Ji S., Almeida E., Guvendiren M. (2019). 3D bioprinting of complex channels within cell-laden hydrogels. Acta Biomater..

[10] Pereira R.F., Sousa A., Barrias C.C., Bayat A., Granja P.L., Bártolo P.J. (2017). Advances in bioprinted cell-laden hydrogels for skin tissue engineering. Biomanuf. Rev..

[11] Derakhshanfar S., Mbeleck R., Xu K., Zhang X., Zhong W., Xing M. (2018). 3D bioprinting for biomedical devices and tissue engineering: A review of recent trends and advances. Bioact. Mater..

[12] Mobaraki M., Ghaffari M., Yazdanpanah A., Luo Y., Mills D.K. (2020). Bioinks and bioprinting: A focused review. Bioprinting.

[13] Taymour R., Kilian D., Ahlfeld T., Gelinsky M., Lode A. (2021). 3D bioprinting of hepatocytes: Core-shell structured co-cultures with fibroblasts for enhanced functionality. Sci. Rep..

[14] Wang X., Li X., Dai X., Zhang X., Zhang J., Xu T., Lan Q. (2018). Coaxial extrusion bioprinted shell-core hydrogel microfibers mimic glioma microenvironment and enhance the drug resistance of cancer cells. Colloids Surf. B Biointerfaces.

[15] Li X., Zhou D., Jin Z., Chen H., Wang X., Zhang X., Xu T. (2020). A coaxially extruded heterogeneous core-shell fiber with Schwann cells and neural stem cells. Regen. Biomater..

[16] Kolesky D.B., Homan K.A., Skylar-Scott M.A., Lewis J.A. (2016). Three-dimensional bioprinting of thick vascularized tissues. Proc. Natl. Acad. Sci. USA.

[17] Lee V.K., Lanzi A.M., Haygan N., Yoo S.-S., Vincent P.A., Dai G. (2014). Generation of Multi-Scale Vascular Network System within 3D Hydrogel using 3D Bio-Printing Technology. Cell. Mol. Bioeng..

[18] Miller J.S., Stevens K.R., Yang M.T., Baker B.M., Nguyen D.-H.T., Cohen D.M., Toro E., Chen A.A., Galie P.A., Yu X. (2012). Rapid casting of patterned vascular networks for perfusable engineered three-dimensional tissues. Nat. Mater..

[19] Gao Q., He Y., Fu J., Liu A., Ma L. (2015). Coaxial nozzle-assisted 3D bioprinting with built-in microchannels for nutrients delivery. Biomaterials.

[20] Gao Q., Liu Z., Lin Z., Qiu J., Liu Y., Liu A., Wang Y., Xiang M., Chen B., Fu J. (2017). 3D Bioprinting of Vessel-like Structures with Multilevel Fluidic Channels. ACS Biomater. Sci. Eng..

[21] Kim M.H., Nam S.Y. (2020). Assessment of coaxial printability for extrusion-based bioprinting of alginate-based tubular constructs. Bioprinting.

[22] Gao G., Kim H., Kim B.S., Kong J.S., Lee J.Y., Park B.W., Chae S., Kim J., Ban K., Jang J. (2019). Tissue-engineering of vascular grafts containing endothelium and smooth-muscle using triple-coaxial cell printing. Appl. Phys. Rev..

[23] Gornall J.L., Terentjev E.M. (2008). Helix-coil transition of gelatin: Helical morphology and stability. Soft Matter.

[24] Ma J., Lin Y., Chen X., Zhao B., Zhang J. (2014). Flow behavior, thixotropy and dynamical viscoelasticity of sodium alginate aqueous solutions. Food Hydrocoll..

[25] Dubbin K., Tabet A., Heilshorn S.C. (2017). Quantitative criteria to benchmark new and existing bio-inks for cell compatibility. Biofabrication.

[26] Jiang T., Munguia-Lopez J.G., Flores-Torres S., Grant J., Vijayakumar S., Leon-Rodriguez A.D., Kinsella J.M. (2017). Directing the Self-assembly of Tumour Spheroids by Bioprinting Cellular Heterogeneous Models within Alginate/Gelatin Hydrogels. Sci. Rep..

[27] Ouyang L., Yao R., Zhao Y., Sun W. (2016). Effect of bioink properties on printability and cell viability for 3D bioplotting of embryonic stem cells. Biofabrication.

[28] Gao T., Gillispie G.J., Copus J.S., Pr A.K., Seol Y.-J., Atala A., Yoo J.J., Lee S.J. (2018). Optimization of gelatin-alginate composite bioink printability using rheological parameters: A systematic approach. Biofabrication.

[29] George M., Abraham T.E. (2006). Polyionic hydrocolloids for the intestinal delivery of protein drugs: Alginate and chitosan—A review. J. Control. Release.

[30] Gudapati H., Yan J., Huang Y., Chrisey D.B. (2014). Alginate gelation-induced cell death during laser-assisted cell printing. Biofabrication.

[31] Steffen W., Ko F.C., Patel J., Lyamichev V., Albert T.J., Benz J., Rudolph M.G., Bergmann F., Streidl T., Kratzsch P. (2017). Discovery of a microbial transglutaminase enabling highly site-specific labeling of proteins. J. Biol. Chem..

[32] Hassell T., Gleave S., Butler M. (1991). Growth inhibition in animal cell culture. The effect of lactate and ammonia. Appl. Biochem. Biotechnol..

[33] Ramdhan T., Ching S.H., Prakash S., Bhandari B. (2019). Time dependent gelling properties of cuboid alginate gels made by external gelation method: Effects of alginate-CaCl_2_ solution ratios and pH. Food Hydrocoll..

[34] Zhou M., Lee B.H., Tan Y.J., Tan L.P. (2019). Microbial transglutaminase induced controlled crosslinking of gelatin methacryloyl to tailor rheological properties for 3D printing. Biofabrication.

[35] Cacopardo L., Ahluwalia A. (2021). Engineering and Monitoring 3D Cell Constructs with Time-Evolving Viscoelasticity for the Study of Liver Fibrosis In Vitro. Bioengineering.

[36] Wu Y., Joseph S., Aluru N.R. (2009). Effect of cross-linking on the diffusion of water, ions, and small molecules in hydrogels. J. Phys. Chem. B.

[37] Visser J., Peters B., Burger T.J., Boomstra J., Dhert W.J.A., Melchels F.P.W., Malda J. (2013). Biofabrication of multi-material anatomically shaped tissue constructs. Biofabrication.

[38] Nie J., Gao Q., Wang Y., Zeng J., Zhao H., Sun Y., Shen J., Ramezani H., Fu Z., Liu Z. (2018). Vessel-on-a-chip with Hydrogel-based Microfluidics. Small.

[39] Wang Z., Ying Z., Bosy-Westphal A., Zhang J., Schautz B., Later W., Heymsfield S.B., Müller M.J. (2010). Specific metabolic rates of major organs and tissues across adulthood: Evaluation by mechanistic model of resting energy expenditure. Am. J. Clin. Nutr..

[40] Kang D., Hong G., An S., Jang I., Yun W.-S., Shim J.-H., Jin S. (2020). Bioprinting of Multiscaled Hepatic Lobules within a Highly Vascularized Construct. Small.

[41] Gillette B.M., Jensen J.A., Tang B., Yang G.J., Bazargan-Lari A., Zhong M., Sia S.K. (2008). In situ collagen assembly for integrating microfabricated three-dimensional cell-seeded matrices. Nat. Mater..

[42] Sarker B., Singh R., Silva R., Roether J.A., Kaschta J., Detsch R., Schubert D.W., Cicha I., Boccaccini A.R. (2014). Evaluation of fibroblasts adhesion and proliferation on alginate-gelatin crosslinked hydrogel. PLoS ONE.

[43] Sarker B., Singh R., Zehnder T., Forgber T., Alexiou C., Cicha I., Detsch R., Boccaccini A.R. (2017). Macromolecular interactions in alginate–gelatin hydrogels regulate the behavior of human fibroblasts. J. Bioact. Compat. Polym..

[44] Xia Y., Zhang X., Bo A., Sun J., Li M. (2018). Sodium citrate inhibits the proliferation of human gastric adenocarcinoma epithelia cells. Oncol. Lett..

[45] Liu W., Zhong Z., Hu N., Zhou Y., Maggio L., Miri A.K., Fragasso A., Jin X., Khademhosseini A., Zhang Y.S. (2018). Coaxial extrusion bioprinting of 3D microfibrous constructs with cell-favorable gelatin methacryloyl microenvironments. Biofabrication.

[46] Benning L., Gutzweiler L., Tröndle K., Riba J., Zengerle R., Koltay P., Zimmermann S., Stark G.B., Finkenzeller G. (2018). Assessment of hydrogels for bioprinting of endothelial cells. J. Biomed. Mater. Res. A.

[47] Pi Q., Maharjan S., Yan X., Liu X., Singh B., van Genderen A.M., Robledo-Padilla F., Parra-Saldivar R., Hu N., Jia W. (2018). Digitally Tunable Microfluidic Bioprinting of Multilayered Cannular Tissues. Adv. Mater. Weinheim..

[48] Cao X., Ashfaq R., Cheng F., Maharjan S., Li J., Ying G., Hassan S., Xiao H., Yue K., Zhang Y.S. (2019). A Tumor-on-a-Chip System with Bioprinted Blood and Lymphatic Vessel Pair. Adv. Funct. Mater..

[49] Kolesky D.B., Truby R.L., Gladman A.S., Busbee T.A., Homan K.A., Lewis J.A. (2014). 3D bioprinting of vascularized, heterogeneous cell-laden tissue constructs. Adv. Mater. Weinheim..

[50] Bertassoni L.E., Cecconi M., Manoharan V., Nikkhah M., Hjortnaes J., Cristino A.L., Barabaschi G., Demarchi D., Dokmeci M.R., Yang Y. (2014). Hydrogel bioprinted microchannel networks for vascularization of tissue engineering constructs. Lab Chip.

[51] Lee H., Cho D.-W. (2016). One-step fabrication of an organ-on-a-chip with spatial heterogeneity using a 3D bioprinting technology. Lab Chip.

[52] Thomas A., Orellano I., Lam T., Noichl B., Geiger M.-A., Amler A.-K., Kreuder A.-E., Palmer C., Duda G., Lauster R. (2020). Vascular bioprinting with enzymatically degradable bioinks via multi-material projection-based stereolithography. Acta Biomater..

[53] Zhu W., Qu X., Zhu J., Ma X., Patel S., Liu J., Wang P., Lai C.S.E., Gou M., Xu Y. (2017). Direct 3D bioprinting of prevascularized tissue constructs with complex microarchitecture. Biomaterials.

[54] Hong S., Kim J.S., Jung B., Won C., Hwang C. (2019). Coaxial bioprinting of cell-laden vascular constructs using a gelatin-tyramine bioink. Biomater. Sci..

