# Supplementary material for: 3D Bioprinting of Prevascularized Full-Thickness Gelatin-Alginate Structures with Embedded Co-Cultures
Source: Bioengineering (Basel). 2022 May 31;9(6):242. doi: 10.3390/bioengineering9060242 (PMC9219913; doi:10.3390/bioengineering9060242)
Supplement: Supplementary file 1 [file bioengineering-09-00242-s001.zip › bioengineering-1706930-supplementary.pdf]

Supplementary Material

Analysis of diffusion process

To analyze the diffusion process, micrographs of the hydrogel were taken that included both the hollow channel and adjacent hydrogel. For data processing, Fiji was used. With this software, the micrographs were converted into a matrix showing the fluorescence intensity for each pixel. For each point on the x-axis, which represented the distance, the mean value was assessed. Using this method, the distance-dependent fluorescence intensity was acquired. As observed in Figure 4, there is a distinct border between the FITC-dextran filled channel and the adjacent hydrogel. This border was also visible in the fluorescence signal and was used as the origin for the distance.

For calculation of the concentration, the fluorescence intensity of FITC-dextran in the hollow channel was determined. According to calibrations, the fluorescence signal showed a linear increase within the used concentration range. Each concentration was calculated according to the measured signal. The concentration change was calculated by comparison of each concentration at the beginning and after 30 min incubation for the given distances.

Rheologic properties after crosslinking and treatment with sodium citrate

Rheologic investigations of the 6 % gelatin 2 % alginate hydrogel (Figure S1) after different phases of crosslinking showed a minor increase in storage modulus compared to non-crosslinked samples at room temperature. However, transglutaminase crosslinking after ionic crosslinking with $CaCl_2$ showed a further increase in mechanical stability. The removal of alginate due to the usage of the chelator sodium citrate and led to a reduction in mechanical stability. This could be connected to the limited availability of functional groups for transglutaminase crosslinking because of steric hindrance of the alginate.

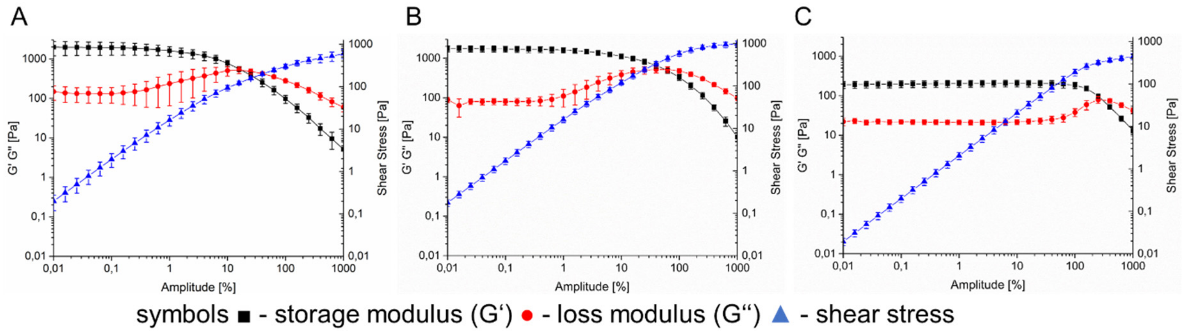

Figure S1. Complex modulus of 6 % gelatin 2 % alginate hydrogel at different phases during crosslinking. A: 10 min treatment with 20 mM $CaCl_2$. B: 60 min treatment with 10 U/ml transglutaminase after $CaCl_2$ treatment. C: 5 min treatment with 100 mM sodium citrate post-crosslinking.

Growth of cell lines in the gelatin–alginate hydrogel

HepG2

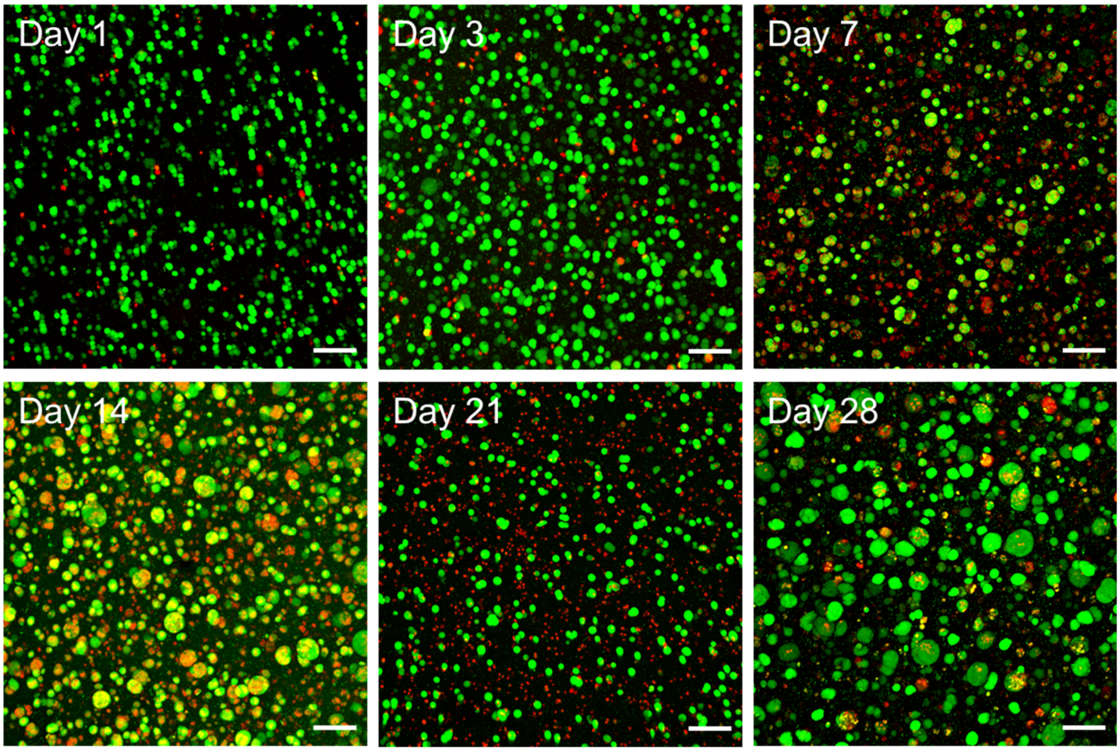

Figure S2. Live–dead staining of HepG2 in 6 % gelatin 2 % alginate hydrogels treated with sodium citrate at various timepoints during cultivation. Green: live cells (calcein AM). Red: dead cells (propidium iodide). Scale Bar. 100 µm

Growth of HepG2 in 6 % gelatin 2 % alginate hydrogels treated with 100 mM sodium citrate for 5 min (Figure S2) showed an initial growth with increasing spheroid size. Up to day 14, an increasing number of dead cells can be observed, especially in spheroids. Afterwards, the regrowth of new HepG2 spheroids could be observed. The reduced amount of dead cells, especially at day 28 could be connected to the degradation of the dead cells' DNA.
Compared with a culture in an untreated hydrogel (Figure S3), spheroids appear smaller and in fewer numbers than in treated hydrogels.

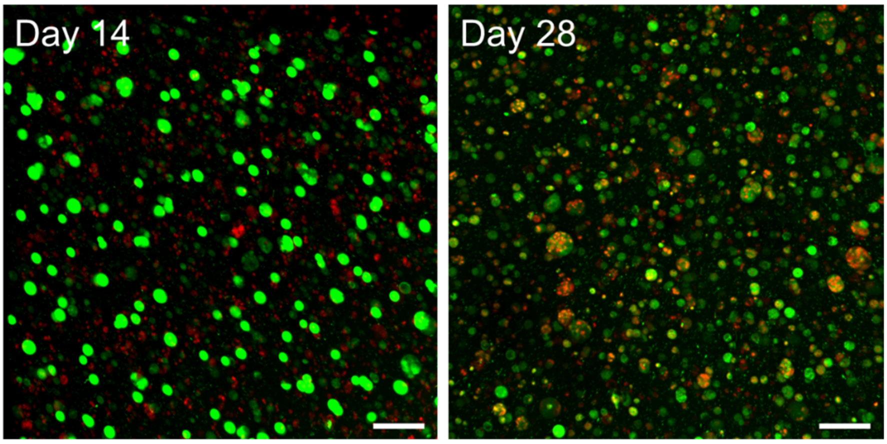

Figure S3. Live–dead staining of HepG2 in a 6 % gelatin 2 % alginate hydrogels without treatment of sodium citrate at various timepoints during cultivation. Green: live cells (calcein AM). Red: dead cells (propidium iodide). Scale Bar. 100 µm

Fibroblasts

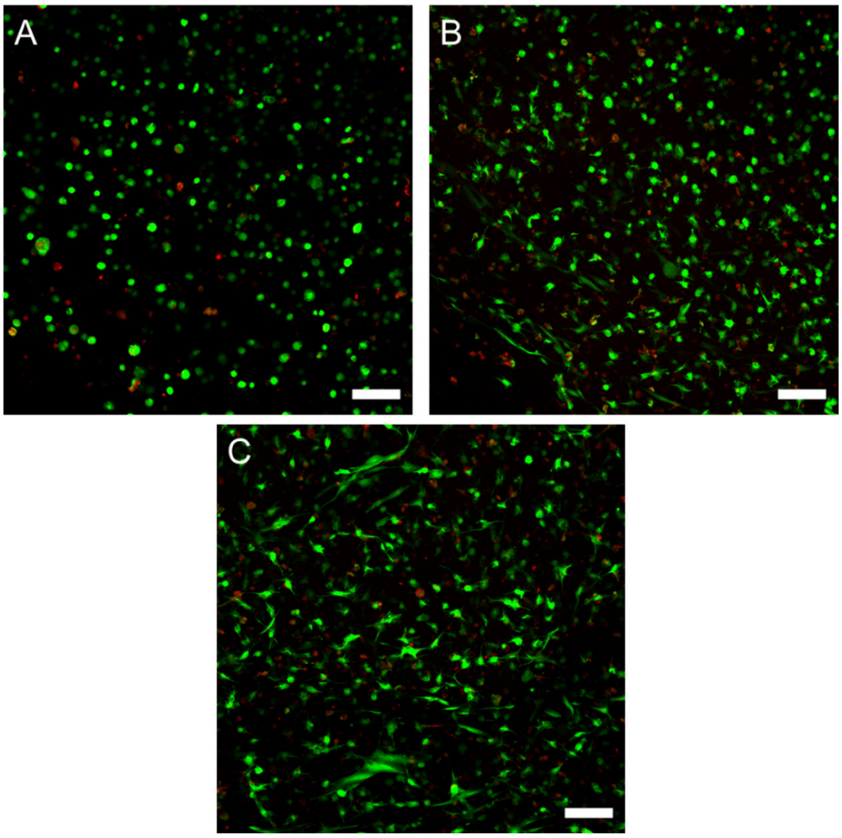

Figure S4: Live–dead staining of fibroblasts in 6 % gelatin 2 % alginate hydrogels after 14 days of culture in gels with different post-crosslinking treatment. A: No treatment with sodium citrate. B: 5 min treatment with 100 mM sodium citrate. C: 10 min treatment with 100 mM sodium citrate. Green: live cells (calcein AM). Red: dead cells (propidium iodide). Scale Bar. 100 µm

The treatment of the hydrogel with sodium citrate showed a strong impact on fibroblasts morphology. While cells remained in a circular shape in untreated hydrogels (Figure S4), an elongation of fibroblasts could be observed in gels treated with sodium citrate. With increasing incubation times for sodium citrate, this elongation is more pronounced.

HUVEC-TERT2

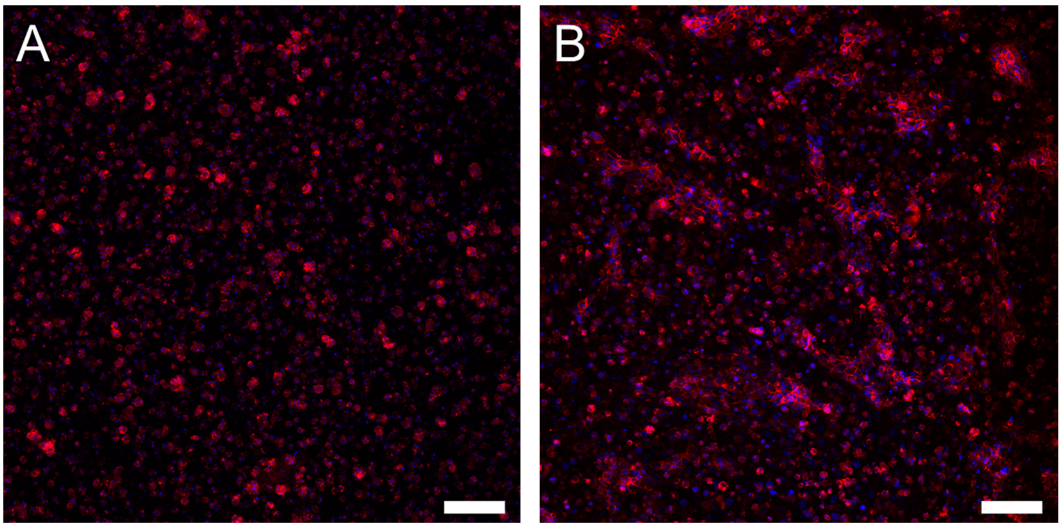

Figure S5. Immunfluorescence staining of HUVEC in a 6 % gelatin 2 % alginate hydrogel after cultivation for two weeks with different post-crosslinking treatment. A: No treatment with sodium citrate. B: 5 min treatment with 100 mM sodium citrate. Blue: Nucleus (DAPI). Red: CD31. Scale Bar: 100 µm

Growth of HUVEC in the 6 % gelatin 2 % alginate hydrogel depended strongly on the sodium citrate treatment after crosslinking. As seen in Figure S5, HUVEC remained mainly isolated, while, after treatment with 100 mM sodium citrate for five minutes, areal growth could be observed through the main cells remain isolated too.

Protein Expression

vWF HUVEC

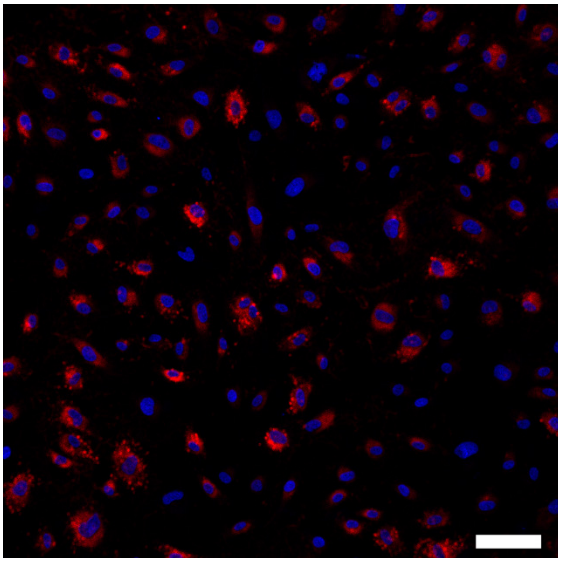

Figure S6. Immunstaining of van Willebrandt Factor of HUVEC. Blue: Nucleus (DAPI). Red: vWF. Scale Bar: 100 µm

Expression of van Willebrandt factor (vWF) could be detected in HUVEC-TERT2 in 2D cell culture (Figure S6).

Collagen I in Co-culture

The cultivation of co-culture consisting of HepG2, HUVEC-TERT2 and fibroblasts in prevascularized constructs showed the expression of collagen I that could positively influence cell growth and vascularization.

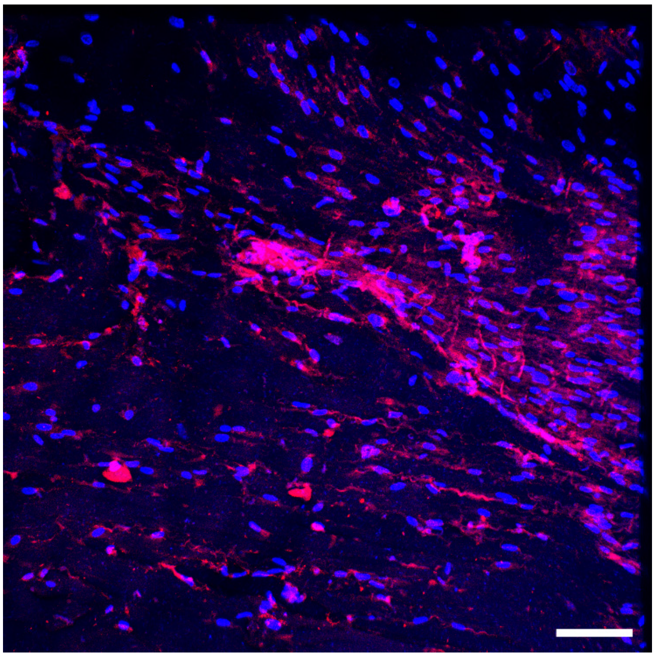

Figure S7. Immunfluorescence Staining of collagen I in a co-culture of HepG2, fibroblasts and HUVEC in prevascularized constructs after 4 weeks. Blue: Nucleus (DAPI). Red: collagen I. Scale Bar: 100 µm

Auxillary Figure

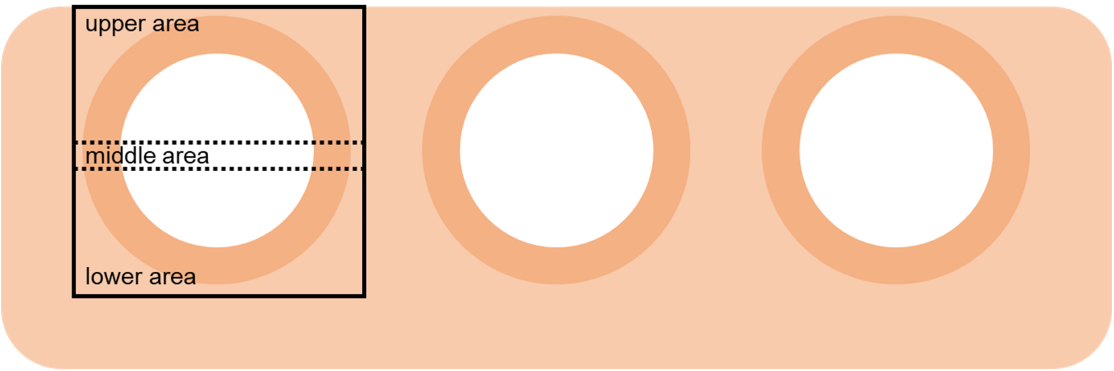

Figure S8. Scheme showing the principal design of the prevascularized construct with the areas that are displayed in Figure 9B,C.
